# Association of Anaemia and Anthropometric Indices Among Chinese Adults: Based on the Sixth China Chronic Disease and Risk Factor Surveillance

**DOI:** 10.3390/nu17193045

**Published:** 2025-09-24

**Authors:** Chuangjia Du, Mei Zhang, Xiao Zhang, Xiaolei Zhu, Chun Li, Zhenping Zhao, Yu Guo, Limin Wang, Xiuyang Li

**Affiliations:** 1 Department of Big Data in Health Sciences and Center for Clinical Big Data and Statistics, Second Affiliated Hospital, College of Medicine, Zhejiang University, Hangzhou 310058, China; 22418058@zju.edu.cn; 2 National Center for Chronic and Noncommunicable Diseases Control and Prevention, Chinese Center for Disease Control and Prevention, Beijing 100050, China; 3 Office of NCD Control and Aging Health, Chinese Center for Disease Control and Prevention, Beijing 100050, China

**Keywords:** anaemia, prevalence, body mass index, waist circumference, waist-to-height ratio, body roundness index

## Abstract

**Background**: Anaemia remains a widespread global public health concern. According to previous research reports, the prevalence rate of anaemia among Chinese adults is lacking. Additionally, the association between anaemia and four common anthropometric indices remains unclear. This study aimed to estimate the prevalence of anaemia and its association with anthropometric indices. **Methods**: The data was from a large, cross-sectional, nationally representative survey which was conducted from August 2018 to June 2019. A total of 190,236 individuals aged 18 years or old were invited, and 159,468 participants with complete data were included in this study. Anaemia was defined as the decrease in adjusted haemoglobin concentrations, <120 g/L for non-pregnant females and <130 g/L for males. Crude and weighted prevalence of anaemia in the overall population and different strata of Chinese adults were calculated. Weighted logistic regression and restricted cubic spline (RCS) were used to evaluate the association between anaemia and four anthropometric indices, including body mass index (BMI), waist circumference (WC), waist-to-height ratio (WHtR), and body roundness index (BRI). **Results**: In China, the weighted anaemia prevalence was 9% (95% CI: 8.5–9.6%), 4.9% (95% CI: 4.4–5.4%), and 13.2% (95% CI: 12.4–13.9%) for the overall population, males, and females, respectively. The weighted prevalence of anaemia was higher among females, rural residents, southwestern residents, and individuals with primary-school-level or lower education than others. The prevalence was highest among young females (14.4%, 95% CI, 13.3–15.5%) and older males (11.8%, 95% CI, 12.4–14.3%). In the fully adjusted logistic regression model, per SD increase in BMI (OR = 0.96, 95% CI: 0.95–0.97), WC (OR = 0.99, 95% CI: 0.98–0.99), WHtR (OR = 0.15, 95% CI: 0.07–0.32), and BRI (OR = 0.90, 95% CI: 0.87–0.94) were associated with a decreased risk of anaemia. Furthermore, the RCS curves depicted L-shaped relationships between the study variables and anaemia (all *p* for nonlinear <0.05). **Conclusions**: The prevalence of anaemia among Chinese adults, especially among young females and underweight older adults, remained unexpectedly high. More attention should be paid to these populations.

## 1. Introduction

Anaemia remains a widespread global public health concern affecting individuals across all age groups [1]. The World Health Organization (WHO) defines anaemia as a decreased blood concentration of haemoglobin (Hb) [2]. Anaemia manifests through a variety of clinical symptoms including generalised weakness, persistent fatigue, and impaired cognitive focus in adults [3]. Moreover, it is associated with an increased risk of multiple adverse health outcomes, such as cardiovascular disease, chronic kidney disease [4], dementia [5], tuberculosis [6], and increased all-cause mortality [7]. In 2021, anaemia affected 1.92 billion people globally with a 24.3% prevalence rate, contributing to 52 million YLDs (Years Lived with Disability), and its prevalence was 9.6% in China, according to the Global Burden of Disease Study 2021 [8].

Previous investigations established that anaemia predominantly affected vulnerable demographics including young children, pregnant and reproductive-age females, particularly in low- and middle-income countries and rural residents [9,10]. Accordingly, anaemia reduction was designated one of the six global nutrition targets (GNTs) of the WHO’s Comprehensive implementation plan on maternal, infant and young child nutrition [11]. In light of the aforementioned evidence, most current research demonstrates a disproportionate focus on children, pregnant and reproductive-age females [12,13]—a trend observed both in China and globally [14,15,16,17]. However, there remains a notable gap in nationwide, real-world investigations of anaemia prevalence among general adults of both genders in China.

Obesity, a prevalent chronic metabolic disorder, affecting approximately 37.0% population around the world [18] and 85 million adults in China [19], was associated with anaemia [20], but the underlying mechanisms remains unclear, with chronic inflammation being a potential contributing factor [21,22]. Body mass index (BMI) is the most commonly used indicator for assessing obesity, and previous studies explored the relationship between BMI and anaemia in limited populations, such as pregnant and pre-pregnancy females [23,24,25]. However, BMI failed to reflect the distribution of body fat [26]. Compared with BMI, other anthropometric indices, such as waist circumference (WC), waist-to-height ratio (WHtR), and body roundness index (BRI) demonstrated superior predictive performance for various diseases [27,28,29,30]. Nevertheless, their associations with anaemia remain poorly understood.

This study aimed to study the association of anaemia and four anthropometric indices, and to report the prevalence of anaemia across different genders, age groups, and townships in China.

## 2. Methods

### 2.1. Survey Design and Populations

The China Chronic Disease and Risk Factor Surveillance (CCDRFS) was first established in 2004 by the National Center for Chronic and Noncommunicable Diseases Control and Prevention (NCNCD). Details of the design, survey methods and protocol of the CCDRFS have been published elsewhere [31]. The sixth CCDRFS was conducted from August 2018 to June 2019 by the NCNCD, and it was a large, cross-sectional, nationally representative survey that covered 31 provincial-level administrative divisions in mainland China [32]. Briefly, the sixth CCDRFS used multistage, stratified, clustered probability sampling to generate a nationally and provincially representative sample. First, 298 out of 605 disease surveillance points (DSPs) were selected using stratified sampling to ensure representativeness across all 31 provinces; in each selected surveillance point, 3 townships or subdistricts were selected by systematic sampling. Then, 2 villages or residentials areas were selected by systematic sampling. Finally, each village or residential area was divided into groups of at least 60 households, and 1 group was selected by simple random sampling. Eligible adult members from 45 households of the selected group who met the inclusion criteria (aged ≥ 18 years, residing at the address for ≥6 months in the past 12 months, not pregnant, and free of serious health conditions) were invited to participate in the survey. The study protocol was approved by the ethics review committee of the NDNCD (approval number: 201819).

### 2.2. Data Collection

The survey included questionnaire interviews, physical examination and laboratory assays [31]. Questionnaire interviews were administered by trained interviewers to obtain information on demographic and socioeconomic characteristics, lifestyle factors, and individual and family history of disease. Physical examination included height, weight, waist circumference, blood pressure, and pulse rate. Height was measured using mechanical anthropometry stadiometers, and weight was measured using electronic body scales which were calibrated on a regular basis according to a standard protocol. Waist circumference was measured in a standing position midway between the lower edge of the costal arch and the upper edge of the iliac crest; each measurement was performed twice to ensure the difference between the two readings was of less than 2 cm, and the two readings were averaged and used for subsequent analysis. Blood pressure was measured on the nondominant arm 3 times consecutively with a 1 min interval between the measurements in a seated position after 5 minutes of rest using an automated device, and the average of the last 2 readings was used for subsequent analysis. Strenuous exercise or physical activity, as well as eating or drinking any beverages (except water), especially those containing caffeine, should be avoided within one hour prior to the measurement. For each participant, blood samples after over 10 h overnight fasting and morning urine samples were collected for laboratory assays, and a 2 h oral glucose tolerance test (OGTT) was performed for participants without a self-reported history of diabetes. Laboratory assays included haemoglobin A_1c_ [HbA_1c_], fasting plasma glucose, triglyceride, total cholesterol [TC], high-density lipoprotein cholesterol [HDL-C], low-density lipoprotein cholesterol [LDL-C], uric acid (UA), serum creatinine (Scr), and triglyceride. The estimated glomerular filtration rate (eGFR) was calculated using the CKD-EPI equation [33]. The haemoglobin concentration was obtained through HemoCue, a rapid portable analyser.

### 2.3. Diagnostic Criteria for Anaemia and Definition of Study Variables

According to the WHO criteria [2], anaemia was defined as the decrease in elevation-adjusted and smoking-adjusted haemoglobin concentrations: <120 g/L for non-pregnant females and <130 g/L for males.

Body mass index (BMI) [34] was calculated as weight in kilograms divided by height in metres squared; both the Chinese BMI standard and the WHO BMI standard were used to define underweight, normal, overweight and obesity. Waist circumference was measured as previously described in data collection, and central obesity was defined as a waist circumference ≥ 90 cm for males and ≥85 cm for females. The waist-to-height ratio (WHtR) was calculated as the waist circumference in centimetres divided by height in centimetres. Body roundness index (BRI) was calculated as 364.2 − 365.5 × √(1 − [waist circumference in centimetres/2π]^2^/[0.5 × height in centimetres]^2^) [35], and was further categorised into four groups according to quartiles.

### 2.4. Associated Factors

Newly detected hypertension was defined as a blood pressure of 140/90 mm Hg or higher at the time of physical examination and without a self-reported history of diagnosed hypertension by physicians in hospitals at the township (community) level or above. Previously diagnosed hypertension was defined as a self-reported physician diagnosis of hypertension and/or if the patient had been taking anti-hypertensive medicine in the past 2 weeks. Newly detected diabetes was defined as a fasting plasma glucose ≥126 mg/dL, a 2 h plasma glucose ≥200 mg/dL in OGTT testing, or an HbA_1c_ ≥ 6.5% [36], without a self-reported history of physician-diagnosed diabetes. Prediabetes was defined as a fasting plasma glucose of 100 to 125 mg/dL, a 2 h plasma glucose of 140 to 199 mg/dL in OGTT testing, or an HbA_1c_ of 5.7% to 6.4% [37]. Dyslipidaemia was defined as total cholesterol ≥ 240 mg/dL, HDL-C ≤ 40 mg/dL, LDL-C ≥ 160 mg/dL, triglyceride ≥ 200 mg/dL, or based on a self-reported physician diagnosis of dyslipidaemia [38]. Hyperuricemia was defined as serum uric acid >420µmol/L. Chronic kidney disease was defined as the presence of impaired kidney function (eGFR <60 mL/min/1.73 m^2^) or albuminuria (urine albumin-to-creatinine ratio of ≥30 mg/g). Broad geographical regions were categorised into 7 sub-regional locations: south (Guangdong, Guangxi, Hainan), east (Shanghai, Jiangsu, Zhejiang, Anhui, Fujian, Jiangxi, Shandong), central (Henan, Hubei, Hunan), north (Beijing, Tianjin, Hebei, Shanxi, Inner Mongolia), northwest (Shaanxi, Gansu, Qinghai, Ningxia), southwest (Chongqing, Sichuan, Guizhou, Yunnan, and Tibet) and northeast (Liaoning, Jilin, Heilongjiang). Fruit/vegetable intake and red meat intake were calculated according to the participants’ recall of the frequency and quantity of consuming fruit/vegetable and red meat over the past 12 months. The participants would be asked whether they had consumed vegetables (or fruit, red meat) in the past 12 months. If so, they would be requested to estimate the frequency of consumption (how many times per day, per week, per month, or per year) and the amount consumed each time.

### 2.5. Statistical Analysis Methods

Categorical variables were described as relative numbers, and continuous variables were described as median (inter-quartile range) since the Kolmogorov–Smirnov test showed all continuous variables were skewedly distributed (*p* ≤ 0.05). Differences between groups were tested using the Wilcoxon rank test or the chi-squared test.

Crude and weighted prevalence of anaemia in the overall population and different strata of Chinese adults were calculated. Differences in proportions between groups were tested using the chi-squared test for crude prevalence, and Rao–Scott chi-squared test for weighted prevalence. Considering differences by gender, age, and township, the overall population was further stratified by gender (male, female), by gender and age groups (young age, middle age and old age), and by gender and township (urban, rural), with differences within each subgroup calculated accordingly. For the weighted prevalence estimations and weighted logistic regression analyses, the STRATA, CLUSTER, and WEIGHT statements in survey package were used to produce nationally representative estimates. The associations of anaemia and all associated factors were evaluated using weighted univariate logistic regression, and covariates were selected if they were reported in the literature or related to anaemia. For each of the study variables, three models were established using weighted multivariable logistic regression to evaluate its association with anaemia, and the restricted cubic spline (RCS) analyses in the fully adjusted logistic model were used to evaluate its nonlinear relationships with anaemia. The number of knots for each spline was set as 4, and all reference points for the study variables were set as their medians.

All data cleaning and analyses were performed using R (version 4.4.2), all tests were two-sided, and *p* ≤ 0.05 was considered statistically significant.

## 3. Results

### 3.1. Study Population

A total of 190,236 eligible adults agreed to participate in the survey, with a response rate of 94.9%. After excluding participants with missing data on cigarette smoking, altitude, haemoglobin concentration, study variables or any other associated factors, 159,468 were included for subsequent analyses among 190,236 participants. The characteristics of the study population are presented in Table 1. The median (25th–75th percentiles) age of study population was 55.7 [46.5, 65.2] years in 2018 to 2019; among these participants, 55.8% were female, and 59.0% resided in rural settings. A total of 16,777 (10.5%) met the diagnostic criteria for anaemia; compared to those without anaemia, these participants had a higher proportion of females, residents living in rural settings, and primary-school-level or lower education, had never smoked or drunk, did not have hypertension or diabetes, and had chronic kidney disease. Participants without anaemia had higher BMI (24.4 kg/m^2^ vs. 23.1 kg/m^2^), WC (84.7 cm vs. 81.0 cm), WHtR (0.5 vs. 0.5), and BRI (4.0 vs. 3.6).

### 3.2. Summary

The overall and gender-specific weighted prevalence of anaemia is shown in Supplementary Table S2. In 2018, the prevalence of anaemia was 9% (95% CI: 8.5–9.6%) for the overall population, 4.9% (95% CI: 4.4–5.4%) for males, and 13.2% (95% CI: 12.4–13.9%) for females, respectively. We observed significant gender disparities in the prevalence of anaemia. Females had a higher prevalence of anaemia in almost every subgroup, except for those aged 60 years or older. Notably, for females, anaemia prevalence was higher among those aged 18–44 years (14.4%, 95% CI 13.3–15.5%), while for males, the prevalence exhibited a consistent increase with advancing age. The participants living in rural settings or in the southwest of China, those who had primary-school-level or lower education, and those with chronic kidney disease had a higher prevalence. Those who had newly detected hypertension, diabetes, dyslipidaemia, and hyperuricemia had a lower prevalence.

The gender- and age-group-specific, and gender- and township-specific weighted prevalence of anaemia are separately shown in Supplementary Tables S4 and S6. Crude prevalence results are shown in Supplementary Tables S1, S3 and S5. Young and older females, living in rural settings, with primary-school-level or lower education had a higher prevalence of anaemia. Notably, for young males and females, the prevalence of anaemia was higher among normal-weight participants, but for middle-aged and older populations, underweight participants had a higher prevalence. Considering different physiological development, the young-age group in females was further divided into 18–30 and 31–44 intervals (Supplemental Table S7).

### 3.3. Factors Related to Anaemia

In the fully adjusted logistic regression model, per SD increase in BMI (OR = 0.96, 95% CI: 0.95–0.97), WC (OR = 0.99, 95% CI: 0.98–0.99), WHtR (OR = 0.15, 95% CI: 0.07–0.32), and BRI (OR = 0.90, 95% CI: 0.87–0.94) were found to be significantly associated with a decreased risk of anaemia. As for categorical study variables, overweight and obese people were less likely to have anaemia than normal-weight counterparts, whether assessed by the Chinese BMI standard or the WHO BMI standard. However, compared with normal weight, underweight status was not found to be associated with anaemia. Central obesity (OR = 0.71, 95% CI: 0.66–0.78), and WHtR over 0.5 (OR = 0.81, 95%: CI 0.74–0.89) were significantly negatively associated with anaemia. Compared with the lowest quartile, those in quartile 3 and quartile 4 of BRI were also less likely to have anaemia (Table 2). The gender- and age-group-specific weighted multivariable logistic regression results are shown in Supplementary Figure S1 and Supplementary Tables S8 and S9. Notably, we observed varying associations across age groups. Underweight middle-aged (OR = 1.75, 95% CI: 1.10–2.79) and older (OR = 2.01, 95% CI: 1.61–2.51) males, as well as older females (OR = 1.66, 95% CI: 1.32–2.10), were more likely to have anaemia, while young males (OR = 0.43, 95% CI: 0.23–0.81) exhibited a lower probability of anaemia compared with normal-weight counterparts. A similar association between other study variables (WC, WHtR and BRI) and anaemia was observed across different age groups of females, but it was only significant in older males. To better appreciate the potential role of obesity in anaemia, the obesity group (assessed by WHO standard) was further categorised into three groups (Class1, 30–34.9 kg/m^2^; Class2, 35–39.9 kg/m^2^; Class3, ≥40 kg/m^2^) (Supplementary Table S10).

**Figure 1:**
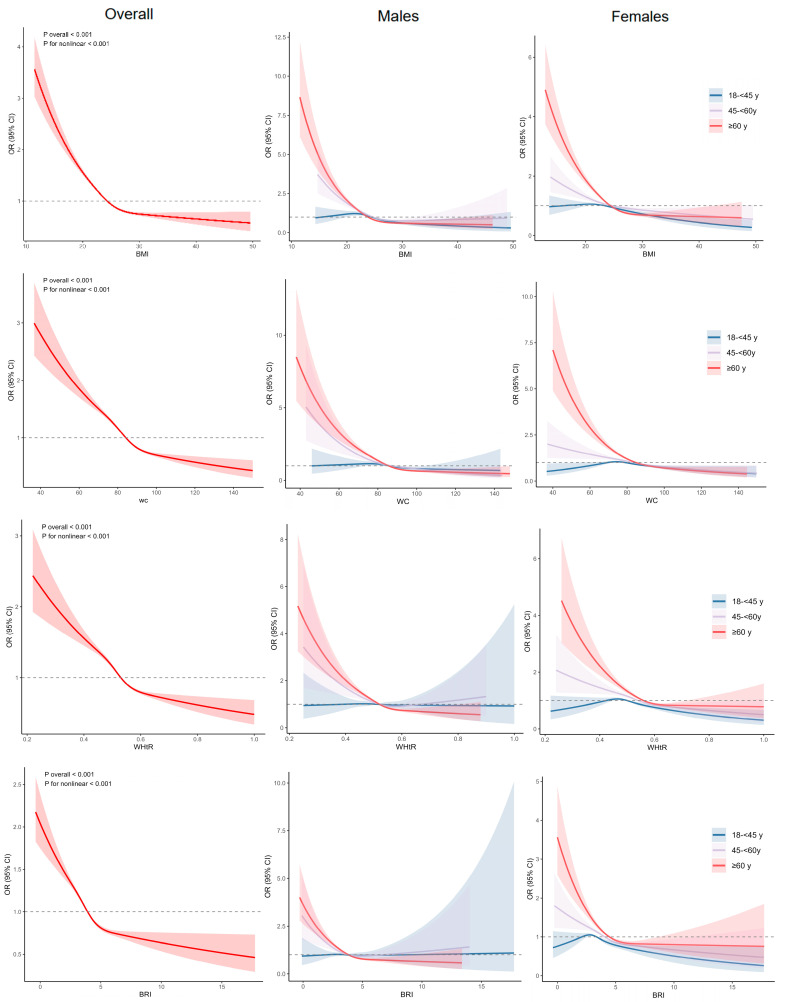
RCS analyses results on the association between the study variables and anaemia.

The RCS plots displayed L-shaped relationships between the study variables and anaemia in the total population and age-group-specific subgroups (Figure 1). The curves depicted a nonlinear relationship between the study variables and anaemia in the total population (all *p* for nonlinear < 0.05). However, the relationship was linear in young, middle-aged males (except BMI) and middle-aged females. We also conducted RCS analyses to evaluate the nonlinear relationships across different subgroups (township, hypertension, and diabetes, Supplementary Figures S1 and S3).

## 4. Discussion

In this study, the anaemia prevalence was 9%, 4.9%, and 13.2% for the overall population, males, and females, respectively, in adults aged 18 years or older in mainland China in 2018. As far as we know, this is the first nationwide, real-world investigation of anaemia among Chinese adults, which provides a detailed description of anaemia prevalence across different genders, townships, and age groups. The CCDRFS was a multistage, stratified, clustered probability sampling survey with standardised and validated protocols, which ensured the population was representative of our estimation.

The findings indicated that the prevalence of anaemia among Chinese adults in 2018 was significantly lower than that reported earlier in 1990 (18.4%) [8]. This decrease in the prevalence of anaemia might be partially attributed to China’s nutrition improvement programs implemented over the past decades [39]. Since dietary iron deficiency remained the primary cause of anaemia in most populations [1], fortification of condiments and seasonings with iron could serve as an effective preventive strategy against anaemia [40]. However, significant disparities in anaemia prevalence were observed across gender and age groups. In this study, the prevalence of anaemia among females was 13.2%, which was comparable to the reported rate of 14.8% among non-pregnant Chinese females aged 18 years or older [41]. However, no available data was found on anaemia prevalence among Chinese males aged 18 years or older in the literature, with only a few studies reporting rates among middle-aged and older males [42,43]. Unlike most studies that specifically examined anaemia in females of reproductive age (15–49 years), this study did not analyse this subgroup separately. Instead, both males and females were categorised by age groups. For females, consistent with previous studies [1], the highest prevalence of anaemia was observed in those aged 18–44 years, primarily attributable to menstrual bleeding and pregnancy-related complications over the reproductive age [44,45,46]. On the other hand, the increased prevalence among females aged 60 years or older might be linked to age-related chronic diseases and inflammation [47,48]. For example, inflammatory bowel disease, as another common cause of anaemia, was reported to have a higher incidence in the elderly population [49]. However, in this study, the anaemia prevalence in females aged 45–59 years was unexpectedly slightly higher than in those aged over 60 years (11.6% vs. 11.4%), diverging from prior findings. This discrepancy might be attributed to menorrhagia, which was common during the perimenopausal period (40–49 years) [50], but we categorised females aged 45–49 years into a middle-aged group, potentially elevating the overall prevalence in this category. Additionally, females aged over 60 years had a higher likelihood of menopause compared with 45–59 counterparts [51]. The prevalence of anaemia increased progressively with advancing age among males, consistent with previous reports [1,47]. Notably, among older adults, the prevalence in males exceeded that in female counterparts (11.8% vs. 11.4%), confirming a trend documented in prior studies [52,53]. Evidence demonstrated that the decline in haemoglobin concentration among older males was more obvious at reduced levels of renal function [54] or at a reduced number of bone marrow erythroid progenitors [55]. For example, in this study, older participants with chronic kidney disease had a higher prevalence of anaemia than young counterparts (19.7% vs. 3.8% in males).

Significant geographic variations were also observed across all genders and age groups. Adults residing in rural areas exhibited substantially higher anaemia prevalence than their urban counterparts, although this disparity was less pronounced among males and middle-aged females. Elevated prevalence was also found in both rural and urban populations of southwestern and southern locations, potentially attributable to poorer economic conditions and uneven healthcare resource distribution in southwestern areas [56,57]. Tea intake, which had been identified as a potential risk factor for anaemia in previous research [58], might be another reason for this geographic difference. Helminth infection (e.g., hookworm infection, ascariasis), which was a common non-nutritional cause of anaemia and more prevalent in southwestern and southern China [12,59], also contributed to such geographic variation. Rural areas of China have been undergoing a rapid urbanisation during the past several decades, but a cross-sectional study in rural China indicated that people in such areas had higher intakes of energy rather than nutrition, which was detrimental to their nutrition status [60]. Additionally, significant educational attainment-related disparities in anaemia prevalence were observed across most age groups for both males and females, consistent with prior findings [8,61]. Interestingly, in this study, income-related disparities in anaemia prevalence were observed exclusively among older and rural populations, reflecting their lower socioeconomic status [1].

The present results demonstrated that across nearly all subgroups, a significantly higher anaemia prevalence was observed in lower BMI, WC, WHtR, BRI groups. Further, detailed associations between anaemia and four anthropometric indices were estimated. In the fully adjusted logistic regression model, overweight and obesity people were less likely to have anaemia than normal-weight counterparts across different genders and age groups. These associations have been investigated and reported in pregnant and reproductive-age females, children, and older populations [25,62,63,64]. The underlying mechanisms of this inverse association remain unclear, though they could be partially explained by a lower likelihood of malnutrition in overweight or obesity individuals. However, some studies reported completely opposite associations [65]. Additionally, underweight status was not found to be significantly associated with anaemia. After age stratification, an age-specific association was observed accordingly; underweight older adults were more likely to have anaemia compared with normal-weight counterparts, and such an association among older adults had been reported before [64]. This might be because older adults were more susceptible to digestive and absorptive impairments caused by gastrointestinal changes, as well as a lower dietary nutrient intake [66,67]. However, young males were less likely to have anaemia; very few studies have reported such an association. Further studies are needed to assess whether this association holds in cohorts. WC and WHtR reflected abdominal obesity [68], which was consistent with the results indicated by BMI groups. BRI took into account height, weight, and waist circumference, providing a more comprehensive assessment of visceral fat distribution. A higher BRI was also associated with a lower risk of anaemia, particularly among females and older males, as a low BRI often indicates malnutrition [69].

This study has several strengths, including, but not limited to, the following: First, this study is the first nationwide, real-world investigation of anaemia among Chinese adults, with strong national representativeness. The present survey provides a detailed description of anaemia prevalence across different genders, townships, and age groups, offering valuable and referable data from China. In addition, the data underwent rigorous quality control and standardisation, ensuring high reliability and quality. Finally, after full adjustment for multiple confounding factors, the associations between four anthropometric indices and anaemia were comprehensively estimated in the general population as well as across gender- and age-group-specific subgroups. These findings are highly representative of the above populations.

This study has several limitations. First, as a cross-sectional analysis, it fails to establish causal relationships or reflect temporal trends in anaemia prevalence in China, nor does it represent the most current epidemiological status; more studies are needed to evaluate long-term trends in anaemia within the Chinese population. Second, dietary iron deficiency remains the leading cause of anaemia [70], but our study defined anaemia solely based on adjusted haemoglobin levels without assessing iron profile (e.g., serum ferritin), and data on dietary iron intake was also unavailable. Third, haemoglobin levels for altitude and smoking status were adjusted per the WHO guidelines, as chronic hypoxia [71] and smoking [72] increase haemoglobin concentrations. However, the altitude adjustment in this guideline was derived from data in Central and South America [2], and its applicability to Chinese populations remains uncertain. These findings showed a higher anaemia prevalence in high-altitude regions (southwestern China) and among some smokers, contradicting the expected haemoglobin-elevating effects of these factors. Prior research suggested that the current definition might overestimate anaemia prevalence in high-altitude populations [73]. Finally, although gender- and age-specific associations between four anthropometric indices and anaemia were comprehensively examined, the results for young males should be interpreted cautiously as the positive effect of underweight was difficult to explain and had not been previously estimated in the literature. Further studies with larger cohorts are needed to validate these findings.

## 5. Conclusions

In 2018, the overall prevalence of anaemia in China was 9%, with 4.9% for males, and 13.2% for females, and significant disparities existed across different genders, age groups, townships, and education levels. Logistic regression revealed that higher BMI, WC, WHtR, and BRI were associated with a lower likelihood of anaemia, particularly among older adults, and underweight older individuals faced a higher anaemia risk. A nonlinear, L-shaped relationship was identified. Our findings highlight that young females and older adults, especially underweight older individuals, remain high-risk groups for anaemia. Targeted interventions should be prioritised for these populations to reduce the burden of anaemia in China.

## Figures and Tables

**Table 1:** Characteristics of the study population in China, 2018.

Characteristics	Total	No Anaemia	Anaemia	*p* Value
Number	159,468	142,691	16,777	
n (%)				
Gender				<0.001
Male	70,477 (44.2)	64,896 (45.5)	5581 (33.3)	
Female	88,991 (55.8)	77,795 (54.5)	11,196 (66.7)	
Township				<0.001
Urban	65,311 (41.0)	59,167 (41.5)	6144 (36.6)	
Rural	94,157 (59.0)	83,524 (58.5)	10,633 (63.4)	
Location in China				<0.001
South	16,780 (10.5)	14,644 (10.3)	2136 (12.7)	
East	41,548 (26.1)	37,506 (26.3)	4042 (24.1)	
Central	19,966 (12.5)	17,900 (12.5)	2066 (12.3)	
North	22,308 (14.0)	20,512 (14.4)	1796 (10.7)	
Northeast	15,050 (9.4)	13,830 (9.7)	1220 (7.3)	
Southwest	23,359 (14.6)	19,671 (13.8)	3688 (22.0)	
Northwest	20,457 (12.8)	18,628 (13.1)	1829 (10.9)	
Education				<0.001
Primary school or lower	78,924 (49.5)	69,262 (48.5)	9662 (57.6)	
Secondary school	48,660 (30.5)	44,188 (31.0)	4472 (26.7)	
High school	20,889 (13.1)	19,192 (13.5)	1697 (10.1)	
College or above	10,995 (6.9)	10,049 (7.0)	946 (5.6)	
Ethnicity				<0.001
Han	140,436 (88.1)	126,485 (88.6)	13,951 (83.2)	
Other	19,032 (11.9)	16,206 (11.4)	2826 (16.8)	
Annual household income, CNY				<0.001
<6000	9132 (5.7)	8044 (5.6)	1088 (6.5)	
6000–11,999	13,625 (8.5)	12,146 (8.5)	1479 (8.8)	
12,000–23,999	20,490 (12.8)	18,312 (12.8)	2178 (13.0)	
≥24,000	81,796 (51.3)	73,823 (51.7)	7973 (47.5)	
Refused/do not know	34,425 (21.6)	30,366 (21.3)	4059 (24.2)	
Cigarette smoking				<0.001
Never	110,873 (69.5)	98,179 (68.8)	12,694 (75.7)	
Former	10,110 (6.3)	9245 (6.5)	865 (5.2)	
Current	38,485 (24.1)	35,267 (24.7)	3218 (19.2)	
Alcohol drinking,				<0.001
No	105,268 (66.0)	93,109 (65.3)	12,159 (72.5)	
Yes	54,200 (34.0)	49,582 (34.7)	4618 (27.5)	
Hypertension				<0.001
No hypertension	94,983 (59.6)	84,060 (58.9)	10,923 (65.1)	
Previously diagnosed	28,413 (17.8)	25,893 (18.1)	2520 (15.0)	
Newly detected	36,072 (22.6)	32,738 (22.9)	3334 (19.9)	
Diabetes				<0.001
No diabetes	60,728 (38.1)	53,537 (37.5)	7191 (42.9)	
Prediabetes	71,469 (44.8)	64,280 (45.0)	7189 (42.9)	
Newly detected	15,773 (9.9)	14,522 (10.2)	1251 (7.5)	
Previously diagnosed	11,498 (7.2)	10,352 (7.3)	1146 (6.8)	
Dyslipidaemia	65,351 (41.0)	60,033 (42.1)	5318 (31.7)	<0.001
Hyperuricemia	17,283 (10.8)	15,628 (11.0)	1655 (9.9)	<0.001
Chronic kidney disease	18,946 (11.9)	16,071 (11.3)	2875 (17.1)	<0.001
Fruit/vegetable intake < 400 g/d	74,694 (46.8)	66,476 (46.6)	8218 (49.0)	<0.001
Red meat intake ≥ 100 g/d	57,634 (36.1)	51,683 (36.2)	5951 (35.5)	0.057
Median [IQR]				
Age, years	55.7 (18.7)	55.8 (18.2)	55.3 (22.5)	0.001
Fasting glucose, mg/dL	100.3 (17.5)	100.6 (17.6)	97.9 (15.8)	<0.001
Glycated haemoglobin A_1c_, %	5.4 (0.6)	5.4 (0.6)	5.3 (0.5)	<0.001
Total cholesterol, mmol/L	4.9 (1.3)	4.9 (1.3)	4.6 (1.3)	<0.001
HDL-C, mmol/L	1.3 (0.5)	1.3 (0.5)	1.4 (0.6)	<0.001
LDL-C, mmol/L	2.9 (1.1)	3.0 (1.2)	2.7 (1.2)	<0.001
Triglyceride, mg/dL	120.5 (89.4)	123.1 (92.1)	104.5 (70.8)	<0.001
Scr, μmol/L	71.0 (22.0)	71.0 (22.0)	67.0 (23.0)	<0.001
BMI, kg/m^2^	24.3 (4.8)	24.4 (4.8)	23.1 (4.7)	<0.001
WC, cm	84.2 (13.8)	84.7 (13.7)	81.0 (13.6)	<0.001
WHtR	0.5 (0.1)	0.5 (0.1)	0.5 (0.1)	<0.001
BRI	3.9 (1.7)	4.0 (1.7)	3.6 (1.6)	<0.001

Data is presented as counts and percentages [n (%)] for categorical variables, and as medians with interquartile ranges [median (IQR)] for continuous variables. Comparisons between anaemia and no-anaemia were performed using the chi-squared test for categorical variables, and the Wilcoxon rank test for continuous variables. BMI, body mass index; WC, waist circumference; WHtR, waist-to-height ratio; BRI, body roundness index; HDL-c, high-density lipoprotein cholesterol; LDL-C, low-density lipoprotein cholesterol; Scr, serum creatinine.

**Table 2:** Logistic regression analyses on study variables and anaemia.

Characteristics	*b*	SE	Wald χ^2^	OR	95% CI	*p* Value
BMI						
Underweight (<18.5)	0.01	0.096	0.004	1.01	(0.83, 1.22)	0.956
Normal (18.5–)				1(Ref)		
Overweight (24–)	−0.24	0.037	41.8	0.79	(0.73, 0.86)	<0.001
Obesity (≥28)	−0.44	0.063	48.8	0.64	(0.57, 0.73)	<0.001
Central obesity						
No				1(Ref)		
Yes	−0.34	0.047	51.7	0.71	(0.66, 0.78)	<0.001
WHtR						
<0.5				1(Ref)		
≥0.5	−0.21	0.454	21.3	0.81	(0.74, 0.89)	<0.001
BRI Quartile ^a^						
Q1				1(Ref)		
Q2	−0.04	0.054	0.5	0.96	(0.87, 1.07)	0.469
Q3	−0.23	0.051	20.6	0.79	(0.71, 0.89)	<0.001
Q4	−0.42	0.062	45.4	0.66	(0.59, 0.73)	<0.001

The logistic model was adjusted for age, gender, township, education, income, cigarette smoking, alcohol drinking, hypertension, diabetes, dyslipidaemia, chronic kidney disease, fruit/vegetable intake, red meat intake, HbA_1c_, HDL-C, LDL-C, Scr, and triglyceride. ^a^ Q1 was <3.12, Q2 was 3.12 to <3.92, Q3 was 3.92 to <4.82, and Q4 was ≥4.82.

## Data Availability

Individual participant data in this study will not be made available publicly. For further detailed data access policy and procedure, please contact jianceshi@ncncd.chinacdc.cn.

## Supplementary Materials

The following supporting information can be downloaded at: https://www.mdpi.com/article/10.3390/nu17193045/s1, Table S1: Crude Prevalence of Anaemia by Gender in Chinese Adults % (95% CI); Table S2: Weighted Prevalence of Anemia by Gender in Chinese Adults % (95% CI); Table S3: Crude Prevalence of Anaemia by Gender and Age Groups in Chinese Adults % (95% CI); Table S4: Weighted Prevalence of Anemia by Gender and Age Groups in Chinese Adults % (95% CI); Table S5: Crude Prevalence of Anaemia by Gender and Township in Chinese Adults % (95% CI); Table S6: Weighted Prevalence of Anaemia by Gender and Township in Chinese Adults % (95% CI); Table S7: Weighted Prevalence of Anaemia by young females in Chinese Adults % (95% CI); Table S8: Logistic Regression Analyses on Males; Table S9: Logistic Regression Analyses on Females; Table S10: Logistic Regression Analyses on detailed obesity category; Figure S1: The gender- and age group-specific logistic regression results after full adjustment; Figure S2: RCS analyses results on the association between study variables and anaemia across different townships; Figure S3: RCS analyses results on the association between study variables and anaemia across different hypertension status; Figure S4: RCS analyses results on the association between study variables and anaemia across different diabetes status.

## Abbreviations

The following abbreviations are used in this manuscript:

WHO	World Health Organization
BMI	Body mass index
WC	Waist circumference
WHtR	Waist-to-height ratio
BRI	Body roundness index
CCDRFS	China Chronic Disease and Risk Factor Surveillance
NCNCD	Noncommunicable Diseases Control and Prevention
HbA_1c_	Haemoglobin A_1c_
HDL-C	High-density lipoprotein cholesterol
LDL-C	Low-density lipoprotein cholesterol
Scr	Serum creatinine
eGFR	Estimated glomerular filtration rate
RCS	Restricted cubic spline
CI	Confidence interval
OR	Odds ratio
