# Supplementary material for: Association of Anaemia and Anthropometric Indices Among Chinese Adults: Based on the Sixth China Chronic Disease and Risk Factor Surveillance
Source: Nutrients. 2025 Sep 24;17(19):3045. doi: 10.3390/nu17193045 (PMC12525547; doi:10.3390/nu17193045)
Supplement: Supplementary file 1 [file nutrients-17-03045-s001.zip › nutrients-3871672-supplementary.pdf]

Supplementary material

Association of Anaemia and Anthropometric Indices among Chinese Adults: Based on the Sixth China Chronic Disease and Risk Factor Surveillance

Table S1: Crude Prevalence of Anaemia by Gender in Chinese Adults % (95% CI)
Table S2: Weighted Prevalence of Anemia by Gender in Chinese Adults % (95% CI)
Table S3: Crude Prevalence of Anaemia by Gender and Age Groups in Chinese Adults % (95% CI)
Table S4: Weighted Prevalence of Anemia by Gender and Age Groups in Chinese Adults % (95% CI)
Table S5: Crude Prevalence of Anaemia by Gender and Township in Chinese Adults % (95%CI)
Table S6: Weighted Prevalence of Anaemia by Gender and Township in Chinese Adults % (95%CI)
Table S7: Weighted Prevalence of Anaemia by young females in Chinese Adults % (95% CI)
Table S8: Logistic Regression Analyses on Males
Table S9: Logistic Regression Analyses on Females
Table S10: Logistic Regression Analyses on detailed obesity category
Figure S1: The gender- and age group-specific logistic regression results after full adjustment
Figure S2: RCS analyses results on the association between study variables and anaemia across different townships
Figure S3: RCS analyses results on the association between study variables and anaemia across different hypertension status
Figure S4: RCS analyses results on the association between study variables and anaemia across different diabetes status

Table S1. Crude Prevalence of Anaemia by Gender in Chinese Adults % (95% CI)

Characteristics	Overall	Male	Female
Overall	10.5(10.4, 10.7)	7.9(7.7, 8.1)	12.6(12.4, 12.8)
Age group			
18-44	12(11.7, 12.3)	4.9 (4.6, 5.2)	17.2 (16.7, 17.7)
45-59	9(8.8, 9.3)	5.5 (5.3, 5.8)	11.6 (11.3, 11.9)
≥60	11.1(10.9, 11.3)	11.4 (11, 11.8)	10.8 (10.5, 11.2)
P for difference	<0.001	<0.001	<0.001
Township			
Urban	9.4(9.2, 9.6)	7 (6.7, 7.3)	11.2 (10.8, 11.5)
Rural	11.3(11.1, 11.5)	8.5 (8.2, 8.8)	13.6 (13.3, 13.9)
P for difference	<0.001	<0.001	<0.001
Location in China			
South	12.7(12.2, 13.2)	10.6 (9.9, 11.3)	14.4 (13.7, 15.1)
East	9.7(9.4, 10)	7 (6.6, 7.4)	11.9 (11.5, 12.3)
Central	10.3(9.9, 10.8)	8.4 (7.8, 9)	11.8 (11.2, 12.4)
North	8.1(7.7, 8.4)	5.2 (4.8, 5.7)	10.2 (9.7, 10.7)
Northeast	8.1(7.7, 8.5)	5.9 (5.4, 6.5)	9.8 (9.1, 10.4)
Southwest	15.8(15.3, 16.3)	13.4 (12.8, 14.1)	17.7 (17.1, 18.4)
Northwest	8.9(8.5, 9.3)	5.1 (4.7, 5.6)	12.2 (11.6, 12.8)
P for difference	<0.001	<0.001	<0.001
Education			
Primary school or lower	12.2(12, 12.5)	10.5 (10.1, 10.8)	13.3 (13, 13.6)
Secondary school	9.2(8.9, 9.4)	6.4 (6.1, 6.7)	12.2 (11.8, 12.6)
High school	8.1(7.8, 8.5)	5.8 (5.4, 6.3)	10.6 (10, 11.2)
College or above	8.6(8.1, 9.1)	5.1 (4.5, 5.7)	11.7 (10.8, 12.5)
P for difference	<0.001	<0.001	<0.001
Ethnicity			
Han	9.9(9.8, 10.1)	7.5 (7.3, 7.7)	11.8 (11.6, 12.1)
Other	14.8(14.3, 15.4)	10.8 (10.2, 11.5)	18 (17.3, 18.8)
P for difference	<0.001	<0.001	<0.001
Annual household income, ¥			
<6000	11.9(11.2, 12.6)	11.1 (10.2, 12)	12.6 (11.7, 13.6)
6000-11999	10.9(10.3, 11.4)	8.7 (8, 9.4)	12.8 (12, 13.5)
12000-23999	10.6(10.2, 11.1)	7.8 (7.3, 8.3)	13 (12.4, 13.7)
≥24000	9.7(9.5, 10)	6.8 (6.5, 7)	12.1 (11.8, 12.4)
Refused/ do not know	11.8(11.5, 12.1)	9.6 (9.1, 10.1)	13.4 (12.9, 13.9)
P for difference	<0.001	<0.001	<0.001
Cigarette smoking			
Never	11.4(11.3, 11.6)	7.4 (7.1, 7.7)	12.6 (12.4, 12.8)
Former	8.6(8, 9.1)	8.4 (7.8, 8.9)	11.4 (8.9, 13.9)
Current	8.4(8.1, 8.6)	8.2 (7.9, 8.4)	11.4 (10.1, 12.6)
P for difference	<0.001	<0.001	0.125
Alcohol drinking,			

Table S1. Crude Prevalence of Anaemia by Gender in Chinese Adults % (95% CI)

Characteristics	Overall	Male	Female
No	11.6(11.4, 11.7)	9.2 (8.9, 9.5)	12.5 (12.3, 12.8)
Yes	8.5(8.3, 8.8)	6.9 (6.7, 7.2)	12.9 (12.4, 13.4)
P for difference	<0.001	<0.001	0.213
Hypertension			
No hypertension	11.5(11.3, 11.7)	7.9 (7.7, 8.2)	14.1 (13.9, 14.4)
Previously diagnosed	8.9(8.5, 9.2)	8.3 (7.8, 8.8)	9.3 (8.8, 9.7)
Newly detected	9.2(8.9, 9.5)	7.7 (7.3, 8.1)	10.8 (10.4, 11.3)
P for difference	<0.001	0.151	<0.001
Diabetes			
No diabetes	11.8(11.6, 12.1)	8 (7.6, 8.3)	14.6 (14.2, 14.9)
Prediabetes	10.1(9.8, 10.3)	7.8 (7.5, 8.1)	12 (11.7, 12.3)
Newly detected	7.9(7.5, 8.4)	7.7 (7.1, 8.3)	8.2 (7.6, 8.8)
Previously diagnosed	10(9.4, 10.5)	8.8 (8, 9.6)	10.8 (10.1, 11.6)
P for difference	<0.001	0.078	<0.001
Dyslipidemia			
No	12.2(12, 12.4)	9.3 (9, 9.6)	14.2 (13.9, 14.5)
Yes	8.1(7.9, 8.3)	6.2 (5.9, 6.4)	10 (9.6, 10.3)
P for difference	<0.001	<0.001	<0.001
Hyperuricemia			
No	10.6(10.5, 10.8)	7.7 (7.5, 7.9)	12.6 (12.4, 12.8)
Yes	9.6(9.1, 10)	8.9 (8.4, 9.4)	12.1 (11, 13.2)
P for difference	<0.001	<0.001	0.401
Chronic kidney diseases			
No	9.9(9.7, 10)	9.1 (8.9, 9.3)	14.3 (14.1, 14.5)
Yes	15.2(14.7, 15.7)	12.7 (11.8, 13.6)	15.9 (14.9, 16.9)
P for difference	<0.001	<0.001	0.002
Fruit/vegetable intake <400g/d			
No	10.1(9.9, 10.3)	7.4 (7.2, 7.7)	12.1 (11.9, 12.4)
Yes	11(10.8, 11.2)	8.4 (8.1, 8.7)	13.1 (12.8, 13.4)
p for difference	<0.001	<0.001	<0.001
Red meat intake ≥100g/d			
No	10.6(10.4, 10.8)	7.9 (7.6, 8.1)	12.5 (12.2, 12.7)
Yes	10.3(10.1, 10.6)	8 (7.7, 8.3)	12.8 (12.4, 13.2)
P for difference	0.057	0.606	0.135
Chinese BMI standard			
Underweight (<18.5)	22.1(21, 23.2)	19 (17.3, 20.7)	19.9 (18.3, 21.4)
Normal (18.5-23.9)	15.1(14.9, 15.4)	10.5 (10.2, 10.9)	15 (14.6, 15.3)
Overweight (24-27.9)	9.9(9.7, 10.1)	5.6 (5.3, 5.9)	11 (10.7, 11.4)
Obesity (≥28)	7.8(7.5, 8.1)	4.2 (3.8, 4.6)	8.6 (8.1, 9)
P for difference	<0.001	<0.001	<0.001
WHO BMI standard			
Underweight (<18.5)	22.1(21, 23.2)	19 (17.3, 20.7)	19.9 (18.3, 21.4)

Table S1. Crude Prevalence of Anaemia by Gender in Chinese Adults % (95% CI)

Characteristics	Overall	Male	Female
Normal (18.5-24.9)	14.2(14, 14.5)	9.7 (9.4, 10)	14.3 (14, 14.6)
Overweight (25-29.9)	9.1(8.9, 9.3)	5 (4.8, 5.3)	10.2 (9.9, 10.6)
Obesity (≥30)	7.7(7.2, 8.1)	4.1 (3.6, 4.7)	8.3 (7.7, 9)
P for difference	<0.001	<0.001	<0.001
Central obesity			
No	14.2(14, 14.4)	9.5 (9.2, 9.8)	14.6 (14.3, 14.9)
Yes	9.1(8.9, 9.3)	5.2 (4.9, 5.4)	9.8 (9.5, 10.1)
P for difference	<0.001	<0.001	<0.001
WHtR group			
<0.5	15.8(15.5, 16.1)	10.5 (10.1, 10.9)	16.4 (15.9, 16.8)
≥0.5	10.7(10.5, 10.8)	6.6 (6.4, 6.8)	11.2 (11, 11.5)
P for difference	<0.001	<0.001	<0.001
BRI group [a]			
Q1	16.2(15.9, 16.5)	10.7 (10.3, 11.2)	16.8 (16.3, 17.3)
Q2	12.5(12.2, 12.8)	7.9 (7.5, 8.3)	13.6 (13.2, 14.1)
Q3	10.6(10.3, 10.9)	6.4 (6.1, 6.8)	11.4 (11, 11.9)
Q4	9.4(9.1, 9.7)	5.8 (5.4, 6.2)	9.5 (9.1, 9.8)
P for difference	<0.001	<0.001	<0.001

[a] Q1 was <3.12, Q2 was 3.12 to <3.92, Q3 was 3.92 to <4.82, Q4 was ⩾4.82

Abbreviations: BMI, body mass index; WC, waist circumference; WHtR, waist-to-height ratio; BRI, body roundness index; CI, confidence interval

Table S2. Weighted Prevalence of Anaemia by Gender in Chinese Adults % (95% CI)

Characteristics	Overall	Male	Female
Overall	9(8.5, 9.6)	4.9(4.4, 5.4)	13.2(12.4, 13.9)
Age group			
18-44	8.6(7.9, 9.3)	2.9 (2.4, 3.5)	14.4 (13.3, 15.5)
45-59	8.2(7.7, 8.8)	4.9 (4.3, 5.6)	11.6 (10.8, 12.3)
≥60	11.6(10.8, 12.5)	11.8 (10.9, 12.7)	11.4 (10.5, 12.3)
P for difference	<0.001	<0.001	<0.001
Township			
Urban	8(7.4, 8.7)	4.2 (3.5, 5)	12 (11.1, 12.9)
Rural	10.1(9.3, 10.8)	5.7 (5, 6.3)	14.4 (13.3, 15.6)
P for difference	<0.001	0.004	<0.001
Location in China			
South	10(9, 11.1)	6.3 (5.4, 7.3)	14 (12.5, 15.5)
East	8(6.9, 9)	3.8 (3.1, 4.4)	12.2 (10.5, 13.8)
Central	9.3(7.9, 10.7)	5 (3.4, 6.6)	13.6 (12.1, 15)
North	8.3(7.5, 9.1)	3.1 (2.5, 3.7)	13.4 (11.9, 14.8)
Northeast	7.7(5.8, 9.7)	4.1 (2.8, 5.5)	11.4 (8.2, 14.5)
Southwest	11.5(9.3, 13.7)	8.2 (5.7, 10.7)	14.9 (12.4, 17.5)
Northwest	9(7.1, 11)	4.5 (3.3, 5.7)	13.7 (10.8, 16.6)
P for difference	0.005	<0.001	0.264
Education			
Primary school or lower	11.6(10.7, 12.5)	8.1 (7.3, 9)	14 (12.8, 15.1)
Secondary school	8.3(7.6, 9.1)	4.4 (3.7, 5.1)	13.3 (12.1, 14.5)
High school	7.6(6.8, 8.3)	3.6 (2.9, 4.2)	13 (11.5, 14.5)
College or above	7.1(6.1, 8.2)	2.9 (2.2, 3.6)	11.4 (9.5, 13.2)
P for difference	<0.001	<0.001	0.08
Ethnicity			
Han	8.9(8.3, 9.4)	4.8 (4.2, 5.3)	13 (12.2, 13.8)
Other	10.5(9.2, 11.9)	6.5 (5.2, 7.7)	14.5 (12.8, 16.1)
P for difference	0.011	0.007	0.087
Annual household income, ¥			
<6000	9.5(8.1, 10.9)	7.3 (5.7, 8.8)	11.9 (9.8, 13.9)
6000-11999	9.6(8.6, 10.6)	6.3 (4.9, 7.6)	13 (11.4, 14.7)
12000-23999	9.2(8.2, 10.2)	5.8 (4.9, 6.7)	12.6 (11, 14.1)
≥24000	8.5(7.9, 9)	4 (3.6, 4.5)	13 (12.1, 13.9)
Refused/ do not know	10.2(9.2, 11.2)	6 (4.8, 7.2)	14.2 (12.8, 15.5)
P for difference	<0.001	<0.001	0.198
Cigarette smoking			
Never	10.5(9.9, 11.2)	4.5 (3.9, 5)	13.1 (12.3, 13.9)
Former	7.4(6.4, 8.5)	6.9 (6, 7.8)	16.7 (8.5, 24.9)
Current	5.3(4.6, 6)	4.9 (4.3, 5.6)	14.4 (11.9, 16.9)
P for difference	<0.001	<0.001	0.382

Table S2. Weighted Prevalence of Anaemia by Gender in Chinese Adults % (95% CI)

Characteristics	Overall	Male	Female
Alcohol drinking,			
No	10.6(9.9, 11.3)	5.5 (4.9, 6.1)	13.1 (12.3, 14)
Yes	6.6(6, 7.3)	4.5 (3.9, 5.1)	13.3 (11.8, 14.9)
P for difference	<0.001	0.001	0.81
Hypertension			
No hypertension	9.5(8.9, 10.1)	4.6 (4, 5.2)	14.1 (13.2, 14.9)
Previously diagnosed	8.6(7.8, 9.3)	7.3 (6.4, 8.1)	9.8 (8.8, 10.8)
Newly detected	7.3(6.6, 8)	5 (4.3, 5.7)	10.6 (9.7, 11.5)
P for difference	<0.001	<0.001	<0.001
Diabetes			
No diabetes	9.5(8.8, 10.1)	4.7 (4, 5.4)	13.6 (12.7, 14.5)
Prediabetes	8.9(8.2, 9.6)	4.9 (4.3, 5.5)	13.7 (12.6, 14.8)
Newly detected	6.6(5.7, 7.4)	5.4 (4.4, 6.3)	8.2 (7, 9.4)
Previously diagnosed	9.3(8.3, 10.3)	7.2 (6.1, 8.3)	11.2 (9.7, 12.7)
P for difference	<0.001	0.005	<0.001
Dyslipidemia			
No	10.7(10, 11.3)	6 (5.3, 6.6)	14.4 (13.5, 15.3)
Yes	6.3(5.7, 6.8)	3.7 (3.2, 4.2)	10.3 (9.4, 11.2)
P for difference	<0.001	<0.001	<0.001
Hyperuricemia			
No	9.5(9, 10.1)	4.8 (4.3, 5.4)	13.3 (12.5, 14.1)
Yes	5.8(5, 6.6)	5.1 (4.2, 6.1)	10.3 (8.5, 12.1)
P for difference	<0.001	0.492	0.004
Chronic kidney disease			
No	8.6(8, 9.2)	4.4 (3.9, 5)	12.9 (12.1, 13.7)
Yes	13.8(12.8, 14.8)	11 (9.9, 12.2)	16.3 (15, 17.6)
P for difference	<0.001	<0.001	<0.001
Fruit/vegetable intake <400g/d			
No	8.8(8.2, 9.4)	4.5 (3.9, 5.1)	13 (12.1, 13.9)
Yes	9.3(8.6, 10)	5.4 (4.8, 6.1)	13.4 (12.3, 14.4)
P for difference	0.165	0.001	0.514
Red meat intake ≥100g/d			
No	9.9(9.3, 10.6)	5 (4.5, 5.5)	13.7 (12.8, 14.6)
Yes	7.8(7.1, 8.5)	4.8 (4, 5.6)	12.2 (11.1, 13.2)
P for difference	<0.001	0.559	0.016
Chinese BMI standard			
Underweight (<18.5)	11.3(9.3, 13.2)	6.9 (5.2, 8.6)	15 (12.1, 17.9)
Normal (18.5-23.9)	10.9(10.2, 11.6)	6.5 (5.8, 7.3)	14.7 (13.8, 15.7)
Overweight (24-27.9)	7.8(7.3, 8.4)	3.9 (3.4, 4.4)	12.3 (11.3, 13.2)
Obesity (≥28)	5.8(5.1, 6.5)	2.8 (2.3, 3.3)	9.5 (8.2, 10.8)
P for difference	<0.001	<0.001	<0.001

Table S2. Weighted Prevalence of Anaemia by Gender in Chinese Adults % (95% CI)

Characteristics	Overall	Male	Female
WHO BMI standard			
Underweight (<18.5)	11.3(9.3, 13.2)	6.9 (5.2, 8.6)	15 (12.1, 17.9)
Normal (18.5-24.9)	10.5(9.8, 11.2)	6 (5.3, 6.7)	14.5 (13.6, 15.5)
Overweight (25-29.9)	7(6.5, 7.6)	3.7 (3.1, 4.2)	11.1 (10.2, 11.9)
Obesity (≥30)	5.8(4.9, 6.7)	2.6 (2, 3.2)	9.7 (7.9, 11.4)
P for difference	<0.001	<0.001	<0.001
Central obesity			
No	10.2(9.6, 10.8)	5.6 (5, 6.3)	14.6 (13.7, 15.4)
Yes	6.8(6.2, 7.5)	3.7 (3.1, 4.2)	10.4 (9.4, 11.3)
P for difference	<0.001	<0.001	<0.001
WHtR group			
<0.5	10.2(9.5, 11)	5.4 (4.8, 6.1)	15 (13.8, 16.1)
≥0.5	8.3(7.7, 8.8)	4.6 (4.1, 5.1)	12 (11.2, 12.8)
P for difference	<0.001	0.002	<0.001
BRI group [a]			
Q1	10.2(9.4, 10.9)	5.4 (4.7, 6.1)	14.9 (13.7, 16)
Q2	9.7(9, 10.4)	5.1 (4.5, 5.8)	14.7 (13.4, 16)
Q3	8(7.3, 8.7)	4.6 (4, 5.3)	11.9 (10.7, 13)
Q4	7.3(6.5, 8.1)	4.1 (3.4, 4.8)	9.9 (8.9, 11)
P for difference	<0.001	0.005	<0.001

a Q1 was <3.12, Q2 was 3.12 to <3.92, Q3 was 3.92 to <4.82, Q4 was ≥4.82

Abbreviations: BMI, body mass index; WC, waist circumference; WHtR, waist-to-height ratio; BRI, body roundness index; CI, confidence interval

Table S3. Crude Prevalence of Anaemia by Gender and Age Groups in Chinese Adults % (95% CI)

	Male			Female		
**Characteristics**	**Young age** [a]	**Middle age** [b]	**Old age** [c]	**Young age** [a]	**Middle age** [b]	**Old age** [c]
Overall	4.9 (4.6, 5.2)	5.5 (5.3, 5.8)	11.4 (11, 11.8)	17.2 (16.7, 17.7)	11.6 (11.3, 11.9)	10.8 (10.5, 11.2)
Township						
Urban	4.4 (3.8, 4.9)	4.9 (4.5, 5.3)	9.9 (9.4, 10.4)	15.3 (14.6, 16.1)	10.6 (10.1, 11.1)	9.4 (8.9, 9.9)
Rural	5.3 (4.8, 5.7)	5.9 (5.5, 6.3)	12.4 (12, 12.9)	18.5 (17.8, 19.2)	12.3 (11.9, 12.8)	12 (11.5, 12.5)
P for difference	0.015	<0.001	<0.001	<0.001	<0.001	<0.001
Location in China						
South	5.7 (4.6, 6.9)	8.2 (7.2, 9.3)	15 (13.7, 16.2)	16.3 (14.8, 17.9)	13.1 (12, 14.2)	14.5 (13.4, 15.7)
East	2.5 (2, 3)	4.3 (3.8, 4.8)	11 (10.3, 11.6)	14.9 (13.9, 15.9)	10.9 (10.2, 11.6)	11.2 (10.6, 11.9)
Central	4 (3, 5)	5.9 (5.1, 6.8)	12.1 (11.1, 13.1)	16.6 (15.1, 18.1)	10.8 (9.9, 11.7)	10.3 (9.4, 11.2)
North	3 (2.1, 3.9)	3.6 (3, 4.2)	7.2 (6.5, 8)	17.2 (15.7, 18.8)	10.1 (9.3, 10.9)	7.1 (6.4, 7.8)
Northeast	4 (2.9, 5.1)	5.3 (4.4, 6.2)	7.3 (6.4, 8.3)	15.9 (14.1, 17.7)	8.7 (7.8, 9.7)	7.9 (7.1, 8.8)
Southwest	10.2 (9, 11.3)	8.9 (7.9, 9.8)	19.4 (18.2, 20.6)	21.1 (19.8, 22.5)	16.3 (15.3, 17.4)	16.6 (15.6, 17.7)
Northwest	4.2 (3.4, 5)	4.3 (3.6, 4.9)	7 (6.1, 7.9)	18.2 (16.9, 19.5)	11.4 (10.5, 12.3)	7 (6.1, 7.9)
P for difference	<0.001	<0.001	<0.001	<0.001	<0.001	<0.001
Education						
Primary school or lower	6.9 (6.1, 7.8)	6.4 (5.8, 6.9)	13.3 (12.8, 13.8)	20.7 (19.7, 21.7)	12.7 (12.3, 13.2)	11.9 (11.5, 12.3)
Secondary school	4.1 (3.6, 4.6)	5.4 (5, 5.8)	9.4 (8.8, 10)	17.5 (16.7, 18.4)	10.8 (10.2, 11.4)	7.6 (6.9, 8.3)
High school	5.2 (4.4, 6)	4.6 (4, 5.2)	7.8 (7, 8.7)	15.8 (14.5, 17.1)	8.8 (8, 9.7)	7.4 (6.4, 8.4)
College or above	4 (3.3, 4.7)	4.3 (3.2, 5.4)	9.2 (7.4, 11)	12.4 (11.4, 13.4)	11.1 (9.4, 12.8)	7.8 (5.5, 10)
P for difference	<0.001	<0.001	<0.001	<0.001	<0.001	<0.001
Ethnicity						
Han	4 (3.6, 4.4)	5.1 (4.8, 5.4)	11 (10.6, 11.4)	15.9 (15.3, 16.5)	11 (10.6, 11.3)	10.5 (10.2, 10.9)
Other	8.7 (7.6, 9.7)	8.6 (7.6, 9.6)	16 (14.6, 17.5)	22.9 (21.6, 24.3)	16.2 (15, 17.3)	14.4 (13.1, 15.7)
P for difference	<0.001	<0.001	<0.001	<0.001	<0.001	<0.001
Annual household income, ¥						
<6000	8 (5.6, 10.3)	7 (5.5, 8.5)	13.3 (12, 14.6)	18.8 (15.8, 21.7)	12.3 (10.6, 14)	11.3 (10.1, 12.5)
6000-11999	5.2 (3.8, 6.6)	5.3 (4.4, 6.3)	11.8 (10.7, 12.9)	19.1 (17, 21.2)	11.6 (10.4, 12.9)	11.1 (10.1, 12.2)
12000-23999	5.6 (4.5, 6.7)	5.5 (4.8, 6.3)	10.8 (9.8, 11.7)	18 (16.4, 19.6)	11.9 (11, 12.9)	11.4 (10.5, 12.4)
≥24000	4.6 (4.1, 5)	5.1 (4.7, 5.5)	9.9 (9.4, 10.4)	16.8 (16.1, 17.4)	11.1 (10.6, 11.5)	9.7 (9.3, 10.2)
Refused/ do not know	4.9 (4.1, 5.7)	6.5 (5.8, 7.2)	13.8 (13, 14.6)	17 (15.9, 18.2)	12.5 (11.8, 13.3)	12.4 (11.7, 13.1)
P for difference	0.005	0.001	<0.001	0.128	0.014	<0.001
Cigarette smoking						
Never	5.4 (4.9, 6)	5.1 (4.6, 5.6)	10.5 (9.9, 11.1)	17.2 (16.7, 17.7)	11.7 (11.3, 12)	10.8 (10.4, 11.1)
Former	5.3 (3.7, 6.8)	5.2 (4.4, 6)	10.3 (9.6, 11.1)	20.7 (10.3, 31.1)	6.7 (2.5, 11)	11.6 (8.6, 14.7)
Current	4.4 (4, 4.9)	5.9 (5.5, 6.2)	12.5 (11.9, 13)	14.9 (10.7, 19.2)	9.3 (7.3, 11.2)	12.1 (10.3, 13.8)
P for difference	0.021	0.038	<0.001	0.48	0.019	0.29
Alcohol drinking,						
No	5.7 (5.1, 6.3)	6 (5.6, 6.5)	12.6 (12, 13.1)	16.8 (16.2, 17.3)	11.7 (11.3, 12.1)	11 (10.7, 11.4)
Yes	4.4 (4, 4.8)	5.2 (4.9, 5.6)	10.2 (9.7, 10.7)	18.8 (17.7, 20)	11.2 (10.4, 12)	9.5 (8.7, 10.4)
P for difference	<0.001	0.007	<0.001	0.002	0.28	0.003
Hypertension						

Table S3. Crude Prevalence of Anaemia by Gender and Age Groups in Chinese Adults % (95% CI)

	Male			Female		
**Characteristics**	**Young age** [a]	**Middle age** [b]	**Old age** [c]	**Young age** [a]	**Middle age** [b]	**Old age** [c]
No hypertension	5.2 (4.8, 5.6)	5.8 (5.4, 6.2)	12.9 (12.3, 13.5)	17.3 (16.8, 17.8)	12.9 (12.5, 13.3)	12 (11.4, 12.5)
Previously diagnosed	1.7 (0.5, 2.9)	5.7 (4.9, 6.4)	9.8 (9.1, 10.4)	17.1 (13.2, 21)	8 (7.2, 8.7)	9.7 (9.1, 10.2)
Newly detected	4.1 (3.3, 4.9)	4.9 (4.3, 5.4)	10.7 (10.1, 11.3)	15.8 (13.9, 17.6)	10.2 (9.5, 10.9)	10.5 (9.9, 11.2)
P for difference	<0.001	0.02	<0.001	0.314	<0.001	<0.001
Diabetes						
No diabetes	5.4 (4.9, 5.9)	5.9 (5.4, 6.4)	12.7 (12, 13.4)	17.6 (16.9, 18.2)	12.9 (12.4, 13.5)	12.6 (11.9, 13.3)
Prediabetes	4.3 (3.8, 4.9)	5.2 (4.8, 5.6)	11.2 (10.7, 11.7)	17.3 (16.3, 18.2)	11.3 (10.8, 11.8)	10.7 (10.2, 11.2)
Newly detected	4.3 (2.9, 5.6)	5.1 (4.3, 5.9)	10.2 (9.3, 11.2)	10.6 (8.3, 12.9)	7.9 (6.9, 8.9)	8 (7.2, 8.8)
Previously diagnosed	4.1 (1.6, 6.6)	6.7 (5.5, 7.9)	10.4 (9.3, 11.5)	13.7 (9.6, 17.8)	10.4 (9, 11.7)	10.8 (9.9, 11.8)
P for difference	0.025	0.013	<0.001	<0.001	<0.001	<0.001
Dyslipidemia						
No	5.5 (5, 6)	6.3 (5.9, 6.7)	13.1 (12.6, 13.5)	17.8 (17.2, 18.4)	13 (12.6, 13.5)	12.5 (12, 12.9)
Yes	4.2 (3.8, 4.7)	4.7 (4.4, 5.1)	8.8 (8.3, 9.3)	15 (14, 16)	9.4 (8.9, 9.9)	9 (8.5, 9.4)
P for difference	<0.001	<0.001	<0.001	<0.001	<0.001	<0.001
Hyperuricemia						
No	4.8 (4.4, 5.2)	5.5 (5.2, 5.8)	10.8 (10.4, 11.2)	17.3 (16.8, 17.9)	11.7 (11.3, 12)	10.6 (10.3, 11)
Yes	5.2 (4.5, 5.9)	5.9 (5.2, 6.6)	14.1 (13.1, 15)	10 (7.3, 12.8)	9.6 (7.9, 11.3)	14.1 (12.5, 15.6)
P for difference	0.274	0.289	<0.001	<0.001	0.034	<0.001
Chronic kidney diseases						
No	4.9 (4.5, 5.2)	5.2 (4.9, 5.5)	10 (9.7, 10.4)	17.1 (16.6, 17.6)	11.4 (11, 11.7)	9.6 (9.2, 9.9)
Yes	5.5 (3.9, 7.2)	9.5 (8.3, 10.8)	17.8 (16.8, 18.9)	18.7 (16.4, 21.1)	13.8 (12.5, 15)	16 (15.1, 16.9)
P for difference	0.458	<0.001	<0.001	0.188	<0.001	<0.001
Fruit/vegetable intake <400g/d						
No	4.6 (4.1, 5)	5.1 (4.7, 5.5)	10.9 (10.5, 11.4)	16.3 (15.6, 17)	11.2 (10.7, 11.6)	10.4 (10, 10.9)
Yes	5.3 (4.8, 5.8)	6 (5.6, 6.4)	11.9 (11.4, 12.4)	18.4 (17.5, 19.2)	12.1 (11.6, 12.6)	11.3 (10.8, 11.7)
P for difference	0.046	0.002	0.009	<0.001	0.005	0.014
Red meat intake ≥100g/d						
No	4.5 (4, 4.9)	5.2 (4.9, 5.6)	11 (10.5, 11.4)	17.9 (17.3, 18.6)	11.5 (11.1, 11.9)	10.7 (10.3, 11.1)
Yes	5.3 (4.8, 5.8)	5.9 (5.5, 6.4)	12.2 (11.6, 12.8)	16 (15.2, 16.8)	11.8 (11.2, 12.4)	11.3 (10.6, 11.9)
P for difference	0.023	0.019	0.002	<0.001	0.429	0.148
Chinese BMI standard						
Underweight (<18.5)	6.8 (4.5, 9)	12.2 (8.9, 15.6)	26.2 (23.7, 28.8)	17 (14.4, 19.5)	16.9 (13.9, 20)	23.5 (21, 25.9)
Normal (18.5-23.9)	6.6 (6, 7.2)	7.2 (6.7, 7.7)	14.5 (14, 15.1)	18.4 (17.7, 19.1)	13.4 (12.9, 14)	13.9 (13.3, 14.5)
Overweight (24-27.9)	3.8 (3.3, 4.4)	4.4 (4, 4.8)	7.5 (7, 8)	17.2 (16.2, 18.1)	10.7 (10.2, 11.2)	8.5 (8, 9)
Obesity (≥28)	2.9 (2.3, 3.5)	3.8 (3.2, 4.3)	5.8 (5, 6.6)	12.6 (11.4, 13.9)	9.1 (8.4, 9.8)	6.1 (5.5, 6.7)
P for difference	<0.001	<0.001	<0.001	<0.001	<0.001	<0.001
WHO BMI standard						
Underweight (<18.5)	6.8 (4.5, 9)	12.2 (8.9, 15.6)	26.2 (23.7, 28.8)	17 (14.4, 19.5)	16.9 (13.9, 20)	23.5 (21, 25.9)
Normal (18.5-24.9)	6 (5.5, 6.6)	6.7 (6.3, 7.1)	13.4 (12.9, 13.9)	18.3 (17.7, 19)	12.9 (12.4, 13.4)	12.9 (12.4, 13.4)
Overweight (25-29.9)	3.7 (3.2, 4.2)	4 (3.6, 4.4)	6.8 (6.3, 7.3)	15.8 (14.9, 16.8)	10.3 (9.8, 10.8)	7.7 (7.2, 8.1)
Obesity (≥30)	2.6 (1.8, 3.4)	4.1 (3.2, 5)	5.9 (4.6, 7.2)	12.2 (10.5, 13.9)	8.4 (7.4, 9.5)	6.1 (5.2, 7)

Table S3. Crude Prevalence of Anaemia by Gender and Age Groups in Chinese Adults % (95% CI)

	Male			Female		
**Characteristics**	**Young age** [a]	**Middle age** [b]	**Old age** [c]	**Young age** [a]	**Middle age** [b]	**Old age** [c]
P for difference	<0.001	<0.001	<0.001	<0.001	<0.001	<0.001
Central obesity						
No	5.6 (5.2, 6.1)	6.3 (5.9, 6.7)	13.8 (13.3, 14.3)	17.9 (17.3, 18.5)	13.1 (12.6, 13.5)	13.6 (13, 14.1)
Yes	3.6 (3.1, 4.1)	4.3 (3.9, 4.7)	6.8 (6.3, 7.3)	15.2 (14.2, 16.1)	9.7 (9.2, 10.1)	8 (7.6, 8.5)
P for difference	<0.001	<0.001	<0.001	<0.001	<0.001	<0.001
WHtR group						
<0.5	5.8 (5.2, 6.4)	7.4 (6.8, 8)	15.9 (15.2, 16.6)	18.2 (17.5, 19)	14.4 (13.6, 15.2)	16.1 (15.2, 17.1)
≥0.5	4.3 (3.8, 4.7)	4.7 (4.4, 5)	9.3 (8.9, 9.7)	16.3 (15.6, 17)	10.7 (10.3, 11.1)	9.7 (9.4, 10.1)
P for difference	<0.001	<0.001	<0.001	<0.001	<0.001	<0.001
BRI group [d]						
Q1	5.8 (5.1, 6.4)	7.4 (6.8, 8.1)	16.5 (15.7, 17.3)	18.4 (17.6, 19.3)	14.9 (14.1, 15.8)	16.7 (15.7, 17.8)
Q2	4.7 (4.1, 5.4)	5.2 (4.7, 5.7)	11.8 (11.1, 12.5)	17.6 (16.5, 18.6)	12.4 (11.8, 13.1)	12.1 (11.3, 12.8)
Q3	4.4 (3.7, 5.1)	4.7 (4.2, 5.2)	8.7 (8.1, 9.4)	16.2 (15, 17.4)	10.7 (10.1, 11.4)	10.3 (9.7, 10.9)
Q4	3.9 (3.1, 4.7)	4.7 (4.1, 5.3)	7.4 (6.8, 8.1)	14.2 (13, 15.5)	9.3 (8.7, 9.9)	8.5 (8, 9)
P for difference	0.001	<0.001	<0.001	<0.001	<0.001	<0.001

[a] young age was defined as 18-44 years, [b] middle age was defined as 45-59 years, [c] old age was defined as 60 years or older, [d] Q1 was <3.12, Q2 was 3.12 to <3.92, Q3 was 3.92 to <4.82, Q4 was ⩾4.82

Abbreviations: BMI, body mass index; WC, waist circumference; WHtR, waist-to-height ratio; BRI, body roundness index; CI, confidence interval

Table S4. Weighted Prevalence of Anaemia by Gender and Age Groups in Chinese Adults % (95% CI)

	Male			Female		
**Characteristics**	**Young age** [a]	**Middle age** [b]	**Old age** [c]	**Young age** [a]	**Middle age** [b]	**Old age** [c]
Overall	2.9 (2.4, 3.5)	4.9 (4.3, 5.6)	11.8 (10.9, 12.7)	14.4 (13.3, 15.5)	11.6 (10.8, 12.3)	11.4 (10.5, 12.3)
Township						
Urban	2.5 (1.7, 3.3)	4.5 (3.7, 5.4)	11.2 (9.8, 12.6)	12.7 (11.4, 14.1)	11.2 (10, 12.4)	10.2 (9, 11.3)
Rural	3.5 (2.8, 4.3)	5.4 (4.5, 6.2)	12.3 (11.3, 13.3)	16.5 (14.8, 18.1)	11.9 (11, 12.8)	12.5 (11.3, 13.6)
P for difference	0.086	0.12	0.159	<0.001	0.347	0.003
Location in China						
South	4.4 (3, 5.7)	7 (5.2, 8.8)	15.3 (13.2, 17.3)	13.6 (11.2, 16)	12.7 (11.2, 14.3)	17.4 (14.5, 20.3)
East	1.8 (1.2, 2.3)	3.7 (2.7, 4.7)	10.4 (8.8, 12.1)	13.3 (11, 15.5)	11.1 (9.7, 12.4)	10.4 (8.5, 12.3)
Central	2.7 (1, 4.5)	5 (2.8, 7.2)	12.5 (10.5, 14.4)	15.5 (13.1, 18)	11 (9.9, 12.2)	11.1 (9.4, 12.9)
North	1.7 (1.1, 2.3)	3.4 (2.8, 4)	7.3 (6, 8.6)	16.5 (14.3, 18.7)	10.8 (9, 12.7)	7.5 (6.2, 8.8)
Northeast	2.5 (1.2, 3.7)	5.4 (3.6, 7.3)	7.4 (4.5, 10.3)	13.6 (8.7, 18.5)	9.7 (7.3, 12)	7.2 (4.7, 9.7)
Southwest	5.4 (2.7, 8.1)	7.1 (4.9, 9.3)	18.8 (15.3, 22.3)	14.8 (11.9, 17.6)	14.1 (11, 17.3)	16.6 (13.8, 19.3)
Northwest	3.3 (2.2, 4.5)	5 (3.3, 6.8)	8.5 (6.3, 10.7)	15.5 (12.4, 18.7)	12.4 (9.2, 15.7)	8.6 (5.1, 12)
P for difference	0.001	0.006	<0.001	0.566	0.101	<0.001
Education						
Primary school or lower	4.3 (3, 5.7)	5.4 (4.5, 6.4)	13.8 (12.6, 15)	18.1 (15.7, 20.5)	12 (11, 13.1)	12.5 (11.5, 13.5)
Secondary school	2.8 (1.9, 3.7)	5.1 (4.3, 5.9)	10 (8.8, 11.2)	14.7 (13, 16.4)	11.4 (10.2, 12.6)	7.9 (6.5, 9.3)
High school	2.9 (2.1, 3.7)	4 (3.2, 4.9)	6.9 (5.6, 8.2)	14.6 (12.6, 16.7)	9.5 (8.1, 11)	7.5 (5.3, 9.7)
College or above	2.4 (1.6, 3.2)	4.3 (3, 5.6)	11.8 (8.5, 15.2)	11.4 (9.3, 13.4)	12.6 (9.5, 15.8)	6.2 (3.7, 8.6)
P for difference	0.058	0.046	<0.001	<0.001	0.075	<0.001
Ethnicity						
Han	2.8 (2.2, 3.4)	4.8 (4.2, 5.5)	11.5 (10.6, 12.5)	14.4 (13.2, 15.6)	11.3 (10.6, 12.1)	11.2 (10.2, 12.1)
Other	4.6 (3.2, 5.9)	6.4 (5.1, 7.7)	15.1 (12.6, 17.6)	14.6 (12.8, 16.4)	13.8 (11.4, 16.3)	15.1 (12.4, 17.7)
P for difference	0.006	0.019	0.005	0.835	0.041	0.003
Annual household income, ¥						
<6000	4 (1.1, 7)	5.2 (3, 7.5)	12.2 (10.2, 14.3)	15.2 (11, 19.4)	9.5 (7.5, 11.6)	9.9 (7.9, 11.8)
6000-11999	4.3 (2.2, 6.3)	4.9 (3.1, 6.8)	10.9 (8.8, 12.9)	15.5 (12.3, 18.6)	10.7 (8.8, 12.5)	11.2 (9.6, 12.9)
12000-23999	3.5 (2.2, 4.8)	5.7 (4.3, 7.2)	11.8 (10.1, 13.6)	13.6 (10.8, 16.3)	10.9 (9.5, 12.3)	12.6 (10.9, 14.3)
≥24000	2.5 (2, 3.1)	4.5 (3.9, 5.1)	10.5 (9.5, 11.5)	14.1 (12.8, 15.3)	11.7 (10.9, 12.5)	10.6 (9.3, 11.9)
Refused/ do not know	3.5 (2.1, 4.9)	5.7 (4.3, 7.1)	14.6 (12.9, 16.2)	15.5 (13.4, 17.5)	12.1 (10.7, 13.6)	13.1 (11.7, 14.5)
P for difference	0.163	0.21	<0.001	0.564	0.253	0.005
Cigarette smoking						
Never	3 (2.4, 3.7)	4.9 (4, 5.7)	10.7 (9.6, 11.9)	14.4 (13.3, 15.5)	11.6 (10.8, 12.3)	11.2 (10.3, 12.2)
Former	3.5 (1.8, 5.3)	5.6 (4.2, 7)	10.6 (9.2, 12.1)	25.3 (4.3, 46.3)	7.2 (1.8, 12.5)	14.4 (9.4, 19.4)
Current	2.8 (2, 3.6)	4.9 (4.2, 5.6)	13.1 (11.9, 14.3)	16.2 (11, 21.4)	11.1 (8.7, 13.6)	15.2 (11.7, 18.7)
P for difference	0.703	0.489	<0.001	0.283	0.382	0.016
Alcohol drinking,						
No	2.4 (1.9, 3)	6 (5.1, 6.9)	12.9 (11.8, 14)	14.3 (13.1, 15.4)	11.7 (10.9, 12.5)	11.7 (10.7, 12.7)
Yes	3.3 (2.5, 4)	4.3 (3.7, 5)	10.7 (9.6, 11.7)	15 (12.6, 17.3)	10.9 (9.6, 12.2)	9.8 (8.4, 11.2)
P for difference	0.036	<0.001	<0.001	0.571	0.233	0.023
Hypertension						

Table S4. Weighted Prevalence of Anaemia by Gender and Age Groups in Chinese Adults % (95% CI)

	Male			Female		
Characteristics	Young age [a]	Middle age [b]	Old age [c]	Young age [a]	Middle age [b]	Old age [c]
No hypertension	3 (2.4, 3.7)	5.3 (4.6, 6.1)	13.2 (12, 14.3)	14.6 (13.4, 15.7)	12.9 (12, 13.7)	13.1 (11.9, 14.3)
Previously diagnosed	3.2 (-0.4, 6.9)	5.1 (4.1, 6.2)	10.7 (9.6, 11.7)	20.3 (14.8, 25.8)	7.6 (6.3, 8.9)	9.8 (8.8, 10.9)
Newly detected	2.5 (1.6, 3.4)	3.9 (3.2, 4.7)	10.8 (9.4, 12.2)	11.2 (9.1, 13.3)	9.9 (8.8, 11.1)	10.9 (9.7, 12.1)
P for difference	0.71	0.007	<0.001	0.001	<0.001	<0.001
Diabetes						
No diabetes	3.3 (2.5, 4.2)	5.2 (4.4, 6)	13.2 (11.8, 14.6)	13.9 (12.7, 15.1)	12.7 (11.7, 13.8)	13.3 (11.9, 14.8)
Prediabetes	2.4 (1.8, 2.9)	4.7 (3.9, 5.5)	11.8 (10.6, 13)	16.4 (14.5, 18.4)	11.3 (10.4, 12.2)	11.4 (10.2, 12.7)
Newly detected	3.2 (1.6, 4.8)	4.2 (2.9, 5.4)	10.3 (8.8, 11.8)	8.8 (6, 11.6)	7.6 (6.2, 9.1)	8 (6.8, 9.3)
Previously diagnosed	3 (0.6, 5.4)	6.4 (4.7, 8.2)	10.2 (8.7, 11.8)	11.9 (5, 18.8)	10.4 (7.9, 12.9)	11.5 (10.1, 12.9)
P for difference	0.111	0.109	0.005	0.002	<0.001	<0.001
Dyslipidemia						
No	3.5 (2.8, 4.3)	5.7 (4.8, 6.5)	13.6 (12.5, 14.8)	15.1 (13.9, 16.3)	13 (12.1, 13.9)	13.4 (12.3, 14.5)
Yes	2.3 (1.7, 2.9)	4.2 (3.6, 4.8)	8.8 (7.9, 9.7)	12 (10.4, 13.6)	9.3 (8.3, 10.2)	9.1 (8.2, 10.1)
P for difference	0.001	<0.001	<0.001	0.001	<0.001	<0.001
Hyperuricemia						
No	2.8 (2.3, 3.3)	4.9 (4.2, 5.6)	11 (10, 12)	14.7 (13.6, 15.8)	11.6 (10.8, 12.3)	11.2 (10.2, 12.1)
Yes	3.3 (2.1, 4.6)	5.1 (4, 6.1)	15.3 (13.8, 16.9)	6.9 (3.4, 10.4)	10.6 (8.1, 13.1)	15.6 (12.8, 18.3)
P for difference	0.364	0.799	<0.001	0.002	0.439	0.001
Chronic kidney disease						
No	2.9 (2.3, 3.5)	4.7 (4, 5.3)	10.1 (9.1, 11)	14.3 (13.2, 15.4)	11.4 (10.6, 12.1)	9.7 (8.8, 10.6)
Yes	3.8 (2.1, 5.6)	7.8 (6.3, 9.3)	19.7 (18, 21.5)	16.7 (13.6, 19.8)	13.8 (11.9, 15.7)	17.4 (15.8, 19)
P for difference	0.257	<0.001	<0.001	0.096	0.008	<0.001
Fruit/vegetable intake <400g/d						
No	2.8 (2.1, 3.4)	4.4 (3.8, 5)	11.1 (10.1, 12.2)	14.2 (13, 15.5)	11.5 (10.6, 12.3)	10.7 (9.6, 11.8)
Yes	3.2 (2.4, 3.9)	5.6 (4.7, 6.5)	12.5 (11.3, 13.6)	14.7 (13.1, 16.3)	11.7 (10.7, 12.6)	12.1 (11, 13.2)
P for difference	0.333	0.005	0.017	0.634	0.729	0.019
Red meat intake ≥100g/d						
No	2.6 (2, 3.1)	4.6 (4, 5.2)	11.3 (10.2, 12.4)	15.8 (14.4, 17.1)	11.6 (10.7, 12.4)	11.2 (10.3, 12.2)
Yes	3.2 (2.4, 4.1)	5.4 (4.3, 6.5)	12.7 (11.5, 14)	12.4 (10.9, 13.8)	11.5 (10.5, 12.6)	12.1 (10.5, 13.7)
P for difference	0.105	0.165	0.044	<0.001	0.989	0.291
Chinese BMI standard						
Underweight (<18.5)	1.8 (0.7, 2.9)	10.8 (6.9, 14.6)	27.9 (23.8, 31.9)	12.9 (9.2, 16.5)	16 (11.8, 20.3)	26.1 (22.3, 29.9)
Normal (18.5-23.9)	3.9 (3, 4.9)	6.2 (5.2, 7.1)	14.7 (13.4, 15.9)	15.1 (13.8, 16.4)	13.4 (12.3, 14.6)	14.8 (13.7, 16)
Overweight (24-27.9)	2.6 (1.9, 3.3)	3.9 (3.3, 4.5)	8.3 (7.2, 9.3)	14.7 (13.1, 16.3)	10.7 (9.9, 11.6)	8.9 (7.9, 9.9)
Obesity (≥28)	1.8 (1.3, 2.3)	4.4 (3.3, 5.4)	5.4 (4.4, 6.3)	11.6 (9.4, 13.8)	9 (7.7, 10.4)	5.1 (4.1, 6)
P for difference	<0.001	<0.001	<0.001	0.038	<0.001	<0.001
WHO BMI standard						
Underweight (<18.5)	1.8 (0.7, 2.9)	10.8 (6.9, 14.6)	27.9 (23.8, 31.9)	12.9 (9.2, 16.5)	16 (11.8, 20.3)	26.1 (22.3, 29.9)
Normal (18.5-24.9)	3.6 (2.8, 4.4)	5.7 (4.9, 6.6)	13.6 (12.5, 14.8)	15.4 (14.1, 16.6)	12.7 (11.8, 13.7)	13.9 (12.8, 14.9)
Overweight (25-29.9)	2.6 (1.9, 3.4)	3.8 (3.2, 4.5)	7.2 (6.3, 8.1)	13.2 (11.7, 14.7)	10.4 (9.5, 11.4)	7.4 (6.4, 8.4)
Obesity (≥30)	1.4 (0.7, 2.1)	5 (3.4, 6.6)	6.3 (4.6, 7.9)	11.6 (8.6, 14.7)	8.7 (7.1, 10.2)	5.6 (4.3, 6.9)

Table S4. Weighted Prevalence of Anaemia by Gender and Age Groups in Chinese Adults % (95% CI)

	Male			Female		
**Characteristics**	**Young age** [a]	**Middle age** [b]	**Old age** [c]	**Young age** [a]	**Middle age** [b]	**Old age** [c]
P for difference	<0.001	<0.001	<0.001	0.029	<0.001	<0.001
Central obesity						
No	3.3 (2.7, 4)	5.4 (4.7, 6.2)	14 (12.9, 15.1)	15.1 (13.9, 16.3)	13.1 (12.2, 14.1)	14.4 (13.3, 15.5)
Yes	2.3 (1.7, 2.9)	4.3 (3.6, 5)	7.6 (6.5, 8.6)	12.4 (10.4, 14.4)	9.5 (8.6, 10.4)	8.3 (7.4, 9.2)
P for difference	0.002	0.003	<0.001	0.023	<0.001	<0.001
WHtR group						
<0.5	3 (2.2, 3.7)	6.2 (5.2, 7.2)	16.3 (15, 17.7)	14.8 (13.4, 16.2)	14.9 (13.5, 16.2)	17.2 (15.5, 18.9)
≥0.5	2.9 (2.3, 3.5)	4.4 (3.8, 5.1)	9.7 (8.7, 10.7)	14 (12.6, 15.4)	10.5 (9.7, 11.3)	10.3 (9.4, 11.2)
P for difference	0.862	<0.001	<0.001	0.359	<0.001	<0.001
BRI group [d]						
Q1	2.9 (2.1, 3.7)	6.2 (5.2, 7.2)	17 (15.5, 18.5)	14.5 (13.2, 15.9)	15.3 (13.8, 16.8)	17.9 (16, 19.9)
Q2	3.3 (2.3, 4.2)	4.8 (4, 5.7)	11.8 (10.6, 13)	16.4 (14.3, 18.4)	12.5 (11.2, 13.7)	12.3 (11, 13.6)
Q3	3.3 (2.4, 4.1)	4.4 (3.6, 5.2)	8.8 (7.7, 10)	13.5 (11.3, 15.6)	10.4 (9.3, 11.4)	11 (9.8, 12.3)
Q4	2.1 (1.3, 2.8)	4.4 (3.6, 5.3)	8.8 (7.5, 10.1)	11.6 (9.4, 13.8)	9.1 (8, 10.2)	9.1 (8, 10.3)
P for difference	0.156	0.002	<0.001	0.01	<0.001	<0.001

a young age was defined as 18-44 years, b middle age was defined as 45-59 years, c old age was defined as 60 years or older, d Q1 was <3.12, Q2 was 3.12 to <3.92, Q3 was 3.92 to <4.82, Q4 was ⩾4.82

Abbreviations: BMI, body mass index; WC, waist circumference; WHtR, waist-to-height ratio; BRI, body roundness index; CI, confidence interval

Table S5. Crude Prevalence of Anaemia by Gender and Township in Chinese Adults % (95%CI)

	Male		Female	
**Characteristics**	**Urban**	**Rural**	**Urban**	**Rural**
Overall	7 (6.7, 7.3)	8.5 (8.2, 8.8)	11.2 (10.8, 11.5)	13.6 (13.3, 13.9)
Age group				
18-44	4.4 (3.8, 4.9)	5.3 (4.8, 5.7)	15.3 (14.6, 16.1)	18.5 (17.8, 19.2)
45-59	4.9 (4.5, 5.3)	5.9 (5.5, 6.3)	10.6 (10.1, 11.1)	12.3 (11.9, 12.8)
≥60	9.9 (9.4, 10.4)	12.4 (12, 12.9)	9.4 (8.9, 9.9)	12 (11.5, 12.5)
P for difference	<0.001	<0.001	<0.001	<0.001
Location in China				
South	9.6 (8.5, 10.7)	11.2 (10.2, 12.1)	13.5 (12.4, 14.6)	15 (14, 15.9)
East	6.1 (5.6, 6.7)	7.8 (7.2, 8.3)	10.6 (10.1, 11.2)	13.1 (12.5, 13.7)
Central	6.3 (5.5, 7.1)	10.2 (9.3, 11)	10.3 (9.5, 11.1)	13.2 (12.3, 14.1)
North	5.1 (4.5, 5.7)	5.3 (4.7, 5.9)	10.1 (9.4, 10.9)	10.3 (9.5, 11)
Northeast	7 (6, 8.1)	5.3 (4.7, 6)	9.9 (9, 10.9)	9.6 (8.8, 10.5)
Southwest	13.3 (12.1, 14.5)	13.5 (12.7, 14.3)	15.8 (14.6, 17)	18.5 (17.7, 19.3)
Northwest	4.5 (3.8, 5.3)	5.4 (4.8, 5.9)	9.9 (8.9, 10.9)	13.3 (12.5, 14)
P for difference	<0.001	<0.001	<0.001	<0.001
Education				
Primary school or lower	10 (9.3, 10.7)	10.7 (10.2, 11.1)	12.3 (11.8, 12.8)	13.7 (13.3, 14)
Secondary school	6 (5.5, 6.4)	6.7 (6.3, 7.1)	10.6 (10, 11.1)	13.8 (13.2, 14.4)
High school	6 (5.4, 6.6)	5.6 (5, 6.3)	9.8 (9.1, 10.5)	12.6 (11.3, 13.8)
College or above	5.4 (4.7, 6.1)	4.2 (3.1, 5.3)	11.2 (10.3, 12.1)	13.6 (11.6, 15.6)
P for difference	<0.001	<0.001	<0.001	0.362
Ethnicity				
Han	6.8 (6.5, 7.1)	8.1 (7.8, 8.3)	10.9 (10.5, 11.2)	12.7 (12.3, 13)
Other	10.4 (8.9, 11.8)	11 (10.2, 11.7)	15.5 (14.1, 16.9)	18.8 (18, 19.7)
P for difference	<0.001	<0.001	<0.001	<0.001
Annual household income, ¥				
<6000	9.4 (7.3, 11.5)	11.4 (10.4, 12.5)	10.1 (8.2, 12.1)	13.2 (12.1, 14.3)
6000-11999	6.9 (5.5, 8.3)	9.1 (8.3, 9.9)	12 (10.4, 13.7)	13 (12.1, 13.8)
12000-23999	7.3 (6.2, 8.4)	8 (7.3, 8.6)	11.6 (10.5, 12.8)	13.5 (12.8, 14.2)
≥24000	6.6 (6.2, 6.9)	7 (6.6, 7.3)	10.7 (10.3, 11.1)	13.7 (13.3, 14.2)
Refused/ do not know	8.2 (7.4, 8.9)	10.4 (9.8, 11)	12.4 (11.7, 13.2)	14 (13.4, 14.6)
P for difference	<0.001	<0.001	<0.001	0.359
Cigarette smoking				
Never	7 (6.5, 7.5)	7.7 (7.3, 8.1)	11.2 (10.8, 11.5)	13.7 (13.4, 14)
Former	7.4 (6.6, 8.2)	9.1 (8.3, 9.8)	12.4 (8.2, 16.7)	10.8 (7.7, 13.9)
Current	6.9 (6.4, 7.3)	8.9 (8.5, 9.2)	10.7 (8.6, 12.8)	11.7 (10.2, 13.3)
P for difference	0.475	<0.001	0.76	0.018
Alcohol drinking,				
No	8.5 (8, 9)	9.6 (9.2, 10)	11.4 (11, 11.7)	13.3 (13, 13.6)
Yes	5.9 (5.5, 6.3)	7.6 (7.3, 7.9)	10.1 (9.4, 10.8)	15.4 (14.6, 16.2)
P for difference	<0.001	<0.001	0.002	<0.001

Table S5. Crude Prevalence of Anaemia by Gender and Township in Chinese Adults % (95%CI)

	Male		Female	
**Characteristics**	**Urban**	**Rural**	**Urban**	**Rural**
Hypertension				
No hypertension	7.1 (6.7, 7.5)	8.4 (8.1, 8.8)	12.3 (11.9, 12.8)	15.5 (15.1, 15.9)
Previously diagnosed	7.6 (6.9, 8.3)	9 (8.3, 9.7)	8.6 (8, 9.3)	9.9 (9.2, 10.5)
Newly detected	6.3 (5.7, 6.9)	8.5 (8, 9)	9.9 (9.2, 10.7)	11.4 (10.8, 12)
P for difference	0.015	0.366	<0.001	<0.001
Diabetes				
No diabetes	7.1 (6.6, 7.6)	8.5 (8, 8.9)	12.9 (12.4, 13.4)	15.7 (15.2, 16.2)
Prediabetes	6.7 (6.3, 7.1)	8.5 (8.1, 8.8)	10.5 (10, 11)	13 (12.6, 13.5)
Newly detected	6.5 (5.6, 7.3)	8.6 (7.8, 9.4)	7.1 (6.3, 8)	9 (8.1, 9.8)
Previously diagnosed	8.5 (7.5, 9.6)	9.2 (8, 10.5)	10.9 (9.8, 11.9)	10.7 (9.6, 11.8)
P for difference	0.003	0.695	<0.001	<0.001
Dyslipidemia				
No	8.4 (7.9, 8.9)	9.8 (9.5, 10.2)	12.7 (12.3, 13.1)	15.3 (14.9, 15.7)
Yes	5.6 (5.2, 6)	6.6 (6.2, 7)	8.8 (8.4, 9.3)	10.8 (10.4, 11.3)
P for difference	<0.001	<0.001	<0.001	<0.001
Hyperuricemia				
No	6.8 (6.5, 7.1)	8.2 (7.9, 8.5)	11.1 (10.8, 11.5)	13.7 (13.4, 14)
Yes	7.7 (7, 8.3)	10 (9.3, 10.6)	11.7 (10.2, 13.2)	12.6 (11, 14.1)
P for difference	0.024	0.005	0.507	0.195
Chronic kidney diseases				
No	6.1 (5.8, 6.4)	7.6 (7.4, 7.9)	10.6 (10.2, 10.9)	13.3 (13, 13.7)
Yes	13.4 (12.2, 14.5)	15.4 (14.4, 16.5)	15.6 (14.5, 16.6)	15.7 (14.8, 16.6)
P for difference	<0.001	<0.001	<0.001	0.033
Fruit/vegetable intake <400g/d				
No	6.7 (6.3, 7.1)	8.1 (7.7, 8.4)	10.7 (10.3, 11.1)	13.5 (13.1, 14)
Yes	7.5 (7, 8)	8.9 (8.5, 9.3)	11.9 (11.3, 12.4)	13.7 (13.3, 14.1)
P for difference	0.013	0.002	<0.001	0.542
Red meat intake ≥100g/d				
No	6.9 (6.5, 7.3)	8.4 (8.1, 8.8)	11 (10.6, 11.4)	13.5 (13.1, 13.8)
Yes	7.2 (6.7, 7.6)	8.6 (8.2, 9)	11.5 (10.9, 12)	14 (13.5, 14.6)
P for difference	0.358	0.56	0.136	0.112
Chinese BMI standard				
Underweight (<18.5)	18 (14.8, 21.2)	19.4 (17.4, 21.5)	17.2 (14.8, 19.7)	21.3 (19.4, 23.3)
Normal (18.5-23.9)	10 (9.4, 10.6)	10.8 (10.4, 11.2)	13.4 (12.9, 13.9)	16.1 (15.6, 16.6)
Overweight (24-27.9)	5.1 (4.7, 5.5)	5.9 (5.6, 6.3)	9.9 (9.4, 10.4)	11.9 (11.5, 12.4)
Obesity (≥28)	4 (3.5, 4.6)	4.3 (3.8, 4.8)	7.6 (7, 8.3)	9.3 (8.7, 9.9)
P for difference	<0.001	<0.001	<0.001	<0.001
WHO BMI standard				
Underweight (<18.5)	18 (14.8, 21.2)	19.4 (17.4, 21.5)	17.2 (14.8, 19.7)	21.3 (19.4, 23.3)
Normal (18.5-24.9)	8.9 (8.4, 9.4)	10.1 (9.7, 10.4)	12.7 (12.2, 13.2)	15.5 (15.1, 15.9)
Overweight (25-29.9)	4.7 (4.4, 5.1)	5.2 (4.9, 5.6)	9.3 (8.8, 9.8)	10.9 (10.5, 11.4)

Table S5. Crude Prevalence of Anaemia by Gender and Township in Chinese Adults % (95%CI)

	Male		Female	
**Characteristics**	**Urban**	**Rural**	**Urban**	**Rural**
Obesity (≥30)	3.9 (3.1, 4.7)	4.4 (3.6, 5.2)	7.4 (6.5, 8.4)	9.1 (8.1, 10)
P for difference	<0.001	<0.001	<0.001	<0.001
Central obesity				
No	8.7 (8.2, 9.1)	10 (9.6, 10.3)	12.9 (12.5, 13.4)	15.8 (15.4, 16.2)
Yes	4.9 (4.5, 5.3)	5.4 (5, 5.8)	8.8 (8.4, 9.2)	10.5 (10.1, 10.9)
P for difference	<0.001	<0.001	<0.001	<0.001
WHtR group				
<0.5	9.9 (9.2, 10.6)	10.8 (10.3, 11.3)	14.6 (13.9, 15.3)	17.8 (17.1, 18.4)
≥0.5	5.9 (5.6, 6.3)	7.1 (6.8, 7.4)	9.9 (9.5, 10.2)	12.2 (11.9, 12.5)
P for difference	<0.001	<0.001	<0.001	<0.001
BRI group [a]				
Q1	10.3 (9.5, 11)	11 (10.4, 11.5)	15.1 (14.3, 15.8)	18.2 (17.5, 18.9)
Q2	6.9 (6.3, 7.4)	8.5 (8, 9)	11.9 (11.2, 12.5)	15 (14.3, 15.6)
Q3	5.7 (5.2, 6.2)	7 (6.5, 7.5)	9.7 (9.1, 10.3)	12.7 (12.1, 13.3)
Q4	5.4 (4.9, 6)	6.1 (5.5, 6.6)	8.6 (8.1, 9.2)	10 (9.5, 10.5)
P for difference	<0.001	<0.001	<0.001	<0.001

[a] Q1 was <3.12, Q2 was 3.12 to <3.92, Q3 was 3.92 to <4.82, Q4 was ≥4.82

Abbreviations: BMI, body mass index; WC, waist circumference; WHtR, waist-to-height ratio; BRI, body roundness index; CI, confidence interval

Table S6. Weighted Prevalence of Anaemia by Gender and Township in Chinese Adults % (95%CI)

	**Male**		**Female**	
**Characteristics**	**Urban**	**Rural**	**Urban**	**Rural**
Overall	4.2 (3.5, 5)	5.7 (5, 6.3)	12 (11.1, 12.9)	14.4 (13.3, 15.6)
Age group				
18-44	2.5 (1.7, 3.3)	3.5 (2.8, 4.3)	12.7 (11.5, 14)	16.5 (14.8, 18.1)
45-59	4.5 (3.7, 5.3)	5.4 (4.5, 6.2)	11.2 (10, 12.3)	11.9 (11, 12.8)
≥60	11.2 (9.8, 12.6)	12.3 (11.3, 13.3)	10.2 (9, 11.3)	12.5 (11.3, 13.6)
P for difference	<0.001	<0.001	0.014	<0.001
Location in China				
South	5.9 (4.4, 7.4)	7.1 (5.2, 8.9)	12.9 (11.9, 14)	15.6 (13.3, 17.9)
East	3.5 (2.8, 4.3)	4.1 (3.2, 4.9)	11.3 (9.9, 12.8)	13.2 (10.8, 15.7)
Central	3.6 (2.2, 5)	6.2 (3.8, 8.6)	11.5 (9.8, 13.2)	15.2 (13.2, 17.2)
North	2.4 (1.6, 3.1)	3.9 (3.4, 4.4)	12.5 (10.1, 14.8)	14.4 (12.6, 16.2)
Northeast	3.8 (2.2, 5.4)	4.6 (2.5, 6.8)	10.8 (7.2, 14.5)	12.1 (6.4, 17.8)
Southwest	7.6 (2.7, 12.4)	8.7 (6.8, 10.5)	14.3 (11.4, 17.3)	15.4 (12.3, 18.6)
Northwest	3.3 (1.7, 4.9)	5.4 (3.9, 6.9)	11 (6.9, 15.2)	15.7 (12.7, 18.7)
P for difference	0.011	<0.001	0.455	0.583
Education				
Primary school or lower	7.6 (6.1, 9.1)	8.4 (7.4, 9.3)	13.7 (12, 15.4)	14.1 (12.8, 15.4)
Secondary school	4 (2.8, 5.2)	4.7 (4, 5.5)	11.8 (10.3, 13.3)	14.7 (12.9, 16.5)
High school	3.6 (2.6, 4.5)	3.5 (2.8, 4.2)	12.2 (10.6, 13.9)	14.5 (11.8, 17.3)
College or above	3.1 (2.3, 4)	2.2 (1.1, 3.3)	10.5 (8.7, 12.3)	15.8 (10.6, 20.9)
P for difference	<0.001	<0.001	0.07	0.831
Ethnicity				
Han	4.2 (3.4, 4.9)	5.5 (4.8, 6.2)	11.9 (11, 12.8)	14.3 (13.1, 15.5)
Other	4.7 (2.6, 6.8)	7.1 (5.7, 8.5)	12.8 (10.3, 15.3)	15.2 (13.4, 16.9)
P for difference	0.627	0.026	0.491	0.399
Annual household income, ¥				
<6000	4.9 (3, 6.8)	8.1 (6.2, 9.9)	8.9 (5.9, 12)	12.7 (10.1, 15.2)
6000-11999	4.6 (2.2, 7)	6.9 (5.5, 8.3)	10.8 (8.2, 13.4)	13.9 (11.9, 16)
12000-23999	5.2 (3.7, 6.7)	6.1 (5, 7.2)	10.8 (8.9, 12.8)	13.3 (11.5, 15.2)
≥24000	3.9 (3.2, 4.5)	4.3 (3.7, 5)	11.8 (10.9, 12.7)	14.9 (13.5, 16.3)
Refused/ do not know	5 (2.9, 7.1)	7 (6, 8.1)	13.3 (11.6, 15.1)	14.9 (13.1, 16.7)
P for difference	0.298	<0.001	0.073	0.314
Cigarette smoking				
Never	3.8 (3.1, 4.5)	5.3 (4.5, 6.2)	11.9 (11.1, 12.8)	14.4 (13.2, 15.5)
Former	6.4 (5.1, 7.6)	7.6 (6.4, 8.7)	17 (9.6, 24.3)	16.5 (5.9, 27.1)
Current	4.2 (3.1, 5.2)	5.6 (4.9, 6.3)	12 (8, 15.9)	16.4 (13, 19.7)
P for difference	0.006	0.003	0.399	0.537
Alcohol drinking,				
No	4.4 (3.6, 5.2)	6.6 (5.9, 7.3)	11.9 (11, 12.9)	14.3 (13.2, 15.5)
Yes	4.1 (3.3, 4.9)	5.1 (4.2, 5.9)	12.2 (10.4, 13.9)	15 (12.8, 17.1)
P for difference	0.325	<0.001	0.789	0.568

Table S6. Weighted Prevalence of Anaemia by Gender and Township in Chinese Adults % (95%CI)

	Male		Female	
**Characteristics**	**Urban**	**Rural**	**Urban**	**Rural**
Hypertension				
No hypertension	3.9 (3.1, 4.7)	5.3 (4.5, 6)	12.6 (11.6, 13.5)	15.8 (14.5, 17)
Previously diagnosed	6.6 (5.2, 8)	8.1 (7.2, 9)	9.5 (8.1, 11)	10.1 (8.9, 11.4)
Newly detected	4 (3.1, 4.9)	6 (5.2, 6.9)	9.9 (8.6, 11.2)	11.1 (10, 12.3)
P for difference	<0.001	<0.001	<0.001	<0.001
Diabetes				
No diabetes	3.9 (2.8, 4.9)	5.5 (4.7, 6.4)	12.2 (11.2, 13.2)	15.2 (13.7, 16.7)
Prediabetes	4.2 (3.4, 5)	5.5 (4.8, 6.3)	12.5 (11.1, 13.9)	14.7 (13.4, 16.1)
Newly detected	4.3 (3.1, 5.6)	6.6 (5.3, 7.9)	7 (5.5, 8.6)	9.3 (7.7, 10.8)
Previously diagnosed	6.7 (5.3, 8.1)	8.1 (6.5, 9.7)	11.7 (9.5, 13.9)	10.6 (8.9, 12.4)
P for difference	0.032	0.037	<0.001	<0.001
Dyslipidemia				
No	5.3 (4.3, 6.3)	6.6 (5.8, 7.3)	12.9 (11.9, 13.9)	15.9 (14.6, 17.3)
Yes	3.1 (2.4, 3.7)	4.5 (3.8, 5.2)	9.6 (8.5, 10.7)	11 (9.9, 12.2)
P for difference	<0.001	<0.001	<0.001	<0.001
Hyperuricemia				
No	4.1 (3.4, 4.8)	5.6 (4.9, 6.3)	12 (11.2, 12.9)	14.6 (13.4, 15.7)
Yes	4.5 (3.2, 5.8)	6.1 (5.1, 7.1)	10.3 (8, 12.7)	10.2 (7.6, 12.9)
P for difference	0.494	0.316	0.178	0.008
Chronic kidney disease				
No	3.7 (3, 4.5)	5.1 (4.5, 5.8)	11.6 (10.8, 12.5)	14.2 (13.1, 15.3)
Yes	9.5 (7.9, 11.2)	12.8 (11.4, 14.3)	16 (14.2, 17.8)	16.6 (14.6, 18.6)
P for difference	<0.001	<0.001	<0.001	0.007
Fruit/vegetable intake <400g/d				
No	3.9 (3.2, 4.7)	5.2 (4.5, 5.9)	11.8 (10.7, 12.9)	14.6 (13.3, 15.9)
Yes	4.6 (3.7, 5.5)	6.1 (5.3, 6.9)	12.2 (10.8, 13.7)	14.3 (12.9, 15.6)
P for difference	0.09	0.013	0.647	0.598
Red meat intake ≥100g/d				
No	4.3 (3.6, 4.9)	5.7 (5, 6.3)	12.6 (11.6, 13.7)	14.7 (13.4, 16)
Yes	4.2 (3.1, 5.2)	5.7 (4.7, 6.7)	11 (9.8, 12.2)	13.8 (12.4, 15.3)
P for difference	0.857	0.946	0.041	0.344
Chinese BMI standard				
Underweight (<18.5)	4.6 (2.8, 6.3)	9.3 (7.1, 11.4)	12.8 (9, 16.6)	17.8 (13.2, 22.4)
Normal (18.5-23.9)	5.9 (4.6, 7.2)	7.1 (6.2, 8)	13.1 (12, 14.2)	16.6 (15.1, 18)
Overweight (24-27.9)	3.6 (2.9, 4.3)	4.3 (3.6, 4.9)	11.1 (9.9, 12.3)	13.4 (12.2, 14.6)
Obesity (≥28)	2.3 (1.7, 2.9)	3.6 (2.9, 4.3)	9.5 (7.7, 11.4)	9.5 (8, 11)
P for difference	<0.001	<0.001	0.006	<0.001
WHO BMI standard				
Underweight (<18.5)	4.6 (2.8, 6.3)	9.3 (7.1, 11.4)	12.8 (9, 16.6)	17.8 (13.2, 22.4)
Normal (18.5-24.9)	5.3 (4.3, 6.4)	6.6 (5.8, 7.5)	12.8 (11.8, 13.9)	16.4 (15, 17.8)
Overweight (25-29.9)	3.4 (2.6, 4.3)	4 (3.4, 4.6)	10.7 (9.5, 11.9)	11.4 (10.3, 12.6)

Table S6. Weighted Prevalence of Anaemia by Gender and Township in Chinese Adults % (95%CI)

	Male		Female	
**Characteristics**	**Urban**	**Rural**	**Urban**	**Rural**
Obesity (≥30)	1.9 (1.3, 2.4)	3.7 (2.4, 5)	9.4 (6.8, 11.9)	10 (8.1, 12)
P for difference	<0.001	<0.001	0.019	<0.001
Central obesity				
No	4.9 (4, 5.8)	7.8 (7, 8.7)	13 (12, 14)	16.3 (15, 17.6)
Yes	3.2 (2.5, 3.9)	4.8 (4.1, 5.4)	9.6 (8.5, 10.8)	11 (9.6, 12.5)
P for difference	<0.001	<0.001	<0.001	<0.001
WHtR group				
<0.5	4.7 (3.7, 5.6)	7.8 (6.9, 8.8)	13.1 (11.9, 14.3)	17.6 (15.8, 19.4)
≥0.5	4 (3.2, 4.7)	6.2 (5.5, 6.9)	11.1 (10.1, 12.2)	12.8 (11.8, 13.9)
P for difference	0.064	<0.001	0.009	<0.001
BRI group [a]				
Q1	4.4 (3.4, 5.4)	8 (6.9, 9)	13 (11.8, 14.2)	17.4 (15.6, 19.3)
Q2	4.7 (3.8, 5.6)	6.7 (5.7, 7.7)	13.4 (11.7, 15.2)	16 (14.4, 17.5)
Q3	4 (3.1, 4.9)	6.2 (5.3, 7.1)	10.5 (9.1, 11.9)	13.1 (11.7, 14.6)
Q4	3.5 (2.5, 4.4)	5.5 (4.7, 6.2)	9.4 (8.1, 10.8)	10.3 (8.9, 11.8)
P for difference	0.09	<0.001	<0.001	<0.001

[a] Q1 was <3.12, Q2 was 3.12 to <3.92, Q3 was 3.92 to <4.82, Q4 was ≥4.82

Abbreviations: BMI, body mass index; WC, waist circumference; WHtR, waist-to-height ratio; BRI, body roundness index; CI, confidence interval

Table S7. Weighted Prevalence of Anaemia by young females in Chinese Adults % (95% CI)

Characteristics	18-30 years	31-44 years
Township		
Urban	8.9 (7.4, 10.5)	16.2 (14.4, 18)
Rural	14.5 (12, 17)	18.1 (16.5, 19.8)
P for difference	<0.001	0.127
Location in China		
South	11.1 (6.7, 15.6)	16.1 (12.9, 19.3)
East	10.5 (7.3, 13.6)	15.7 (13.5, 17.9)
Central	12.7 (9.6, 15.9)	18.1 (14.9, 21.4)
North	12.1 (8.8, 15.3)	20.6 (18.4, 22.7)
Northeast	7.3 (2.4, 12.1)	18.4 (13.4, 23.4)
Southwest	12.2 (8, 16.4)	16.8 (12.9, 20.6)
Northwest	14.8 (9.9, 19.7)	16.2 (13.3, 19)
P for difference	0.499	0.354
Education		
Primary school or lower	15.4 (9.9, 21)	18.8 (16.5, 21.1)
Secondary school	11.8 (8.9, 14.7)	16.9 (15.4, 18.5)
High school	12 (9, 15)	18.1 (15.4, 20.9)
College or above	9.7 (7.2, 12.3)	14.1 (11, 17.2)
P for difference	0.235	0.062
Ethnicity		
Han	11.5 (9.8, 13.1)	17 (15.7, 18.3)
Other	10.9 (8.3, 13.4)	18 (16.2, 19.9)
P for difference	0.687	0.35
Annual household income, ¥		
<6000	11.5 (3.9, 19.1)	18.2 (14.3, 22.1)
6000-11999	11.1 (5.2, 16.9)	18.8 (15.9, 21.7)
12000-23999	11.5 (7, 16)	15.4 (12.7, 18)
≥24000	11.2 (9.4, 13)	16.6 (15.2, 18)
Refused/ do not know	12 (8.9, 15.1)	18.6 (16.1, 21.1)
P for difference	0.991	0.172
Cigarette smoking		
Never	11.4 (9.8, 12.9)	17 (15.8, 18.3)
Former	35.7 (-13.8, 85.1)	18.2 (4.1, 32.4)
Current	9.8 (3.7, 15.8)	21.2 (10.5, 32)
P for difference	0.18	0.585
Alcohol drinking,		
No	11.2 (9.5, 12.8)	17.1 (15.8, 18.5)
Yes	12.4 (9.1, 15.7)	17 (14.5, 19.6)
P for difference	0.501	0.956
Hypertension		
No hypertension	11.7 (10.1, 13.2)	17.3 (16, 18.6)
Previously diagnosed	7.1 (1.1, 13.1)	21.8 (15.7, 27.9)

Table S7. Weighted Prevalence of Anaemia by young females in Chinese Adults % (95% CI)

Characteristics	18-30 years	31-44 years
Newly detected	5.4 (2, 8.8)	13.5 (11.2, 15.9)
P for difference	0.012	0.008
Diabetes		
No diabetes	11.3 (9.7, 12.9)	16.7 (15.3, 18.1)
Prediabetes	13 (9.3, 16.7)	18.4 (16.6, 20.3)
Newly detected	5.7 (0.8, 10.6)	11.2 (8, 14.5)
Previously diagnosed	0.1 (-0.1, 0.2)	16.3 (8, 24.6)
P for difference	0.07	0.037
Dyslipidemia		
No	12.2 (10.5, 13.9)	17.8 (16.4, 19.2)
Yes	7.7 (5.3, 10.1)	14.9 (13, 16.7)
P for difference	0.006	0.006
Hyperuricemia		
No	11.6 (10, 13.2)	17.3 (16.1, 18.6)
Yes	6.8 (1.7, 11.9)	7 (3.9, 10.2)
P for difference	0.153	<0.001
Chronic kidney diseases		
No	11.5 (9.9, 13)	16.9 (15.6, 18.1)
Yes	9.5 (4, 15)	21.2 (17.2, 25.1)
P for difference	0.532	0.018
Fruit/vegetable intake <400g/d		
No	10.4 (8.8, 11.9)	17.4 (15.9, 19)
Yes	12.7 (10.1, 15.3)	16.6 (14.7, 18.4)
p for difference	0.092	0.458
Red meat intake ⩾100g/d		
No	12.9 (11, 14.9)	18.2 (16.6, 19.7)
Yes	9.2 (7.1, 11.4)	15.3 (13.7, 17)
P for difference	0.013	0.011
Chinese BMI standard		
Underweight (<18.5)	12.8 (8, 17.5)	13.2 (7.8, 18.6)
Normal (18.5-23.9)	12.3 (10.4, 14.3)	18 (16.5, 19.5)
Overweight (24-27.9)	10.1 (7, 13.2)	17.3 (15.6, 19.1)
Obesity (≥28)	7.8 (4.4, 11.1)	14.1 (11.9, 16.3)
P for difference	0.173	0.019
WHO BMI standard		
Underweight (<18.5)	12.8 (8, 17.5)	13.2 (7.8, 18.6)
Normal (18.5-24.9)	12.6 (10.7, 14.5)	18 (16.5, 19.4)
Overweight (25-29.9)	7.7 (4.9, 10.4)	16.3 (14.7, 17.9)
Obesity (≥30)	8.2 (3.1, 13.3)	14.3 (11.5, 17.1)
P for difference	0.044	0.036
Central obesity		
No	12 (10.3, 13.7)	18.1 (16.7, 19.5)

Table S7. Weighted Prevalence of Anaemia by young females in Chinese Adults % (95% CI)

**Characteristics**	**18-30 years**	**31-44 years**
Yes	9.1 (6, 12.2)	14.5 (12.6, 16.3)
P for difference	0.138	<0.001
WHtR group		
<0.5	12.6 (10.6, 14.5)	17.6 (16.1, 19.2)
≥0.5	9.5 (7.5, 11.5)	16.7 (15, 18.3)
P for difference	0.026	0.365
BRI group [a]		
Q1	11.9 (10.1, 13.8)	17.9 (16.4, 19.5)
Q2	13.2 (9.6, 16.8)	18.3 (16.1, 20.5)
Q3	9.2 (5.1, 13.3)	16 (13.9, 18.1)
Q4	7.3 (4.5, 10.2)	14 (11.4, 16.6)
P for difference	0.116	0.017

Table S8. Logistic Regression Analyses on Males

	Male overall		Young age		Middle age		Old age	
**Characteristics**	**OR（95% CI）**	***P* value**	**OR（95% CI）**	***P* value**	**OR（95% CI）**	***P* value**	**OR（95% CI）**	***P* value**
**BMI per SD**	**0.94 (0.92, 0.96)**	**< 0.001**	**0.99 (0.95, 1.02)**	**0.483**	**0.95 (0.92, 0.99)**	**0.006**	**0.88 (0.86, 0.90)**	**< 0.001**
Chinese BMI standard								
Underweight (<18.5)	1.13 (0.91, 1.41)	0.27	**0.41 (0.22, 0.77)**	**0.005**	**1.66 (1.04, 2.66)**	**0.034**	**1.91 (1.53, 2.39)**	**< 0.001**
Normal (18.5-23.9)	1(Ref)		1(Ref)		1(Ref)		1(Ref)	
Overweight (24-27.9)	**0.68 (0.58, 0.79)**	**< 0.001**	0.78 (0.54, 1.11)	0.159	**0.69 (0.57, 0.85)**	**< 0.001**	**0.60 (0.52, 0.69)**	**< 0.001**
Obesity (≥28)	**0.59 (0.47, 0.76)**	**< 0.001**	**0.58 (0.34, 0.98)**	**0.043**	0.80 (0.59, 1.08)	0.148	**0.40 (0.31, 0.50)**	**< 0.001**
***P* for trend**		**< 0.001**		**0.028**		**0.008**		**< 0.001**
WHO BMI standard								
Underweight (<18.5)	1.20 (0.96, 1.49)	0.105	**0.43 (0.23, 0.81)**	**0.008**	**1.75 (1.10, 2.79)**	**0.019**	**2.01 (1.61, 2.51)**	**< 0.001**
Normal (18.5-24.9)	1(Ref)		1(Ref)		1(Ref)		1(Ref)	
Overweight (25-29.9)	**0.73 (0.62, 0.86)**	**< 0.001**	0.88 (0.62, 1.26)	0.487	**0.74 (0.61, 0.91)**	**0.003**	**0.58 (0.50, 0.68)**	**< 0.001**
Obesity (≥30)	**0.68 (0.50, 0.91)**	**0.01**	**0.52 (0.28, 0.97)**	**0.041**	1.02 (0.67, 1.55)	0.938	**0.54 (0.38, 0.78)**	**0.001**
***P* for trend**		**< 0.001**		0.086		0.067		**< 0.001**
**WC per SD**	**0.98 (0.98, 0.99)**	**< 0.001**	1.00 (0.99, 1.02)	0.667	**0.99 (0.98, 1.00)**	**0.006**	**0.97 (0.96, 0.97)**	**< 0.001**
**Central obesity**								
No	1(Ref)		1(Ref)		1(Ref)		1(Ref)	
Yes	**0.76 (0.66, 0.88)**	**< 0.001**	0.84 (0.58, 1.20)	0.341	0.93 (0.76, 1.13)	0.44	**0.58 (0.50, 0.67)**	**< 0.001**
**WHtR per SD**	**0.29 (0.09, 0.92)**	**0.036**	6.07 (0.78, 47.13)	0.084	0.34 (0.05, 2.12)	0.244	**0.01 (0.00, 0.04)**	**< 0.001**
**WHtR group**								
<0.5	1(Ref)		1(Ref)		1(Ref)		1(Ref)	
≥0.5	0.88 (0.76, 1.02)	0.086	1.29 (0.92, 1.81)	0.139	**0.83 (0.69, 0.99)**	**0.043**	**0.64 (0.57, 0.72)**	**< 0.001**
**BRI per SD**	**0.94 (0.88, 0.99)**	**0.029**	1.08 (0.98, 1.19)	0.136	0.95 (0.87, 1.04)	0.295	**0.81 (0.76, 0.86)**	**< 0.001**
**Quartile [a]**								
Q1	1(Ref)		1(Ref)		1(Ref)		1(Ref)	
Q2	0.95 (0.80, 1.12)	0.541	1.37 (0.94, 2.01)	0.106	0.88 (0.70, 1.11)	0.279	**0.73 (0.63, 0.84)**	**< 0.001**
Q3	0.88 (0.73, 1.07)	0.199	1.47 (0.96, 2.26)	0.076	0.85 (0.67, 1.08)	0.176	**0.55 (0.47, 0.65)**	**< 0.001**
Q4	**0.78 (0.64, 0.94)**	**0.011**	1.00 (0.60, 1.66)	0.995	0.88 (0.67, 1.17)	0.397	**0.53 (0.44, 0.65)**	**< 0.001**
***P* for trend**		**0.015**		0.393		0.328		**< 0.001**

Analyses were fully adjusted for age, gender, township, education, income, cigarette smoking, alcohol drinking, hypertension, diabetes, dyslipidemia, chronic kidney diseases, fruit/vegetable intake, red meat intake, HbA_{1c}, HDL-C, LDL-C, Scr, and triglyceride

Abbreviations: BMI, body mass index; WC, waist circumference; WHtR, waist-to-height ratio; BRI, body roundness index; CI, confidence interval; OR, odds ratio

[a]Q1 was <3.12, Q2 was 3.12 to <3.92, Q3 was 3.92 to <4.82, Q4 was ≥4.82

Table S9. Logistic Regression Analyses on Females

	Female overall		Young age		Middle age		Old age	
**Characteristics**	**OR（95% CI）**	***P* value**	**OR（95% CI）**	***P* value**	**OR（95% CI）**	***P* value**	**OR（95% CI）**	***P* value**
**BMI per SD**	**0.97 (0.96, 0.98)**	**< 0.001**	**0.98 (0.96, 1.00)**	**0.019**	**0.96 (0.94, 0.98)**	**< 0.001**	**0.90 (0.88, 0.92)**	**< 0.001**
Chinese BMI standard								
Underweight (<18.5)	0.99 (0.77, 1.27)	0.92	0.92 (0.64, 1.32)	0.652	1.28 (0.89, 1.85)	0.186	**1.59 (1.26, 2.01)**	**< 0.001**
Normal (18.5-23.9)	1(Ref)		1(Ref)		1(Ref)		1(Ref)	
Overweight (24-27.9)	**0.85 (0.77, 0.94)**	**0.002**	0.87 (0.74, 1.02)	0.089	**0.82 (0.73, 0.93)**	**0.001**	**0.64 (0.56, 0.73)**	**< 0.001**
Obesity (≥28)	**0.67 (0.58, 0.78)**	**< 0.001**	**0.71 (0.56, 0.90)**	**0.004**	**0.70 (0.60, 0.83)**	**< 0.001**	**0.38 (0.31, 0.46)**	**< 0.001**
***P* for trend**		**< 0.001**		**0.005**		**< 0.001**		**< 0.001**
WHO BMI standard								
Underweight (<18.5)	0.99 (0.77, 1.28)	0.967	0.92 (0.64, 1.32)	0.653	1.33 (0.92, 1.92)	0.126	**1.66 (1.32, 2.10)**	**< 0.001**
Normal (18.5-24.9)	1(Ref)		1(Ref)		1(Ref)		1(Ref)	
Overweight (25-29.9)	**0.78 (0.70, 0.86)**	**< 0.001**	**0.77 (0.66, 0.91)**	**0.002**	**0.84 (0.75, 0.95)**	**0.004**	**0.58 (0.51, 0.67)**	**< 0.001**
Obesity (≥30)	**0.69 (0.57, 0.85)**	**< 0.001**	**0.73 (0.53, 1.00)**	**0.048**	**0.71 (0.57, 0.89)**	**0.003**	**0.45 (0.34, 0.59)**	**< 0.001**
***P* for trend**		**< 0.001**		**< 0.001**		**< 0.001**		**< 0.001**
**WC per SD**	**0.99 (0.98, 0.99)**	**< 0.001**	0.99 (0.98, 1.00)	0.076	**0.99 (0.98, 0.99)**	**< 0.001**	**0.97 (0.96, 0.97)**	**< 0.001**
**Central obesity**								
No	1(Ref)		1(Ref)		1(Ref)		1(Ref)	
Yes	**0.72 (0.65, 0.80)**	**< 0.001**	**0.75 (0.63, 0.90)**	**0.001**	**0.78 (0.70, 0.88)**	**< 0.001**	**0.62 (0.55, 0.70)**	**< 0.001**
**WHtR per SD**	**0.17 (0.07, 0.39)**	**< 0.001**	**0.22 (0.06, 0.78)**	**0.02**	**0.12 (0.04, 0.32)**	**< 0.001**	**0.04 (0.02, 0.08)**	**< 0.001**
**WHtR group**								
<0.5	1(Ref)		1(Ref)		1(Ref)		1(Ref)	
≥0.5	**0.80 (0.72, 0.90)**	**< 0.001**	**0.82 (0.70, 0.96)**	**0.015**	**0.77 (0.68, 0.87)**	**< 0.001**	**0.63 (0.55, 0.73)**	**< 0.001**
**BRI per SD**	**0.91 (0.87, 0.95)**	**< 0.001**	**0.92 (0.86, 0.98)**	**0.012**	**0.90 (0.86, 0.95)**	**< 0.001**	**0.86 (0.82, 0.89)**	**< 0.001**
**Quartile** [a]								
Q1	1(Ref)		1(Ref)		1(Ref)		1(Ref)	
Q2	0.97 (0.85, 1.10)	0.658	0.99 (0.82, 1.18)	0.882	**0.85 (0.73, 0.98)**	**0.029**	**0.72 (0.60, 0.85)**	**< 0.001**
Q3	**0.78 (0.68, 0.89)**	**< 0.001**	**0.77 (0.62, 0.97)**	**0.023**	**0.72 (0.61, 0.84)**	**< 0.001**	**0.66 (0.55, 0.78)**	**< 0.001**
Q4	**0.66 (0.57, 0.75)**	**< 0.001**	**0.67 (0.53, 0.84)**	**< 0.001**	**0.68 (0.58, 0.80)**	**< 0.001**	**0.53 (0.44, 0.63)**	**< 0.001**
***P* for trend**		**< 0.001**		**< 0.001**		**< 0.001**		**< 0.001**

Analyses were fully adjusted for age, gender, township, education, income, cigarette smoking, alcohol drinking, hypertension, diabetes, dyslipidemia, chronic kidney diseases, fruit/vegetable intake, red meat intake, HbA_{1c}, HDL-C, LDL-C, Scr, and triglyceride

Abbreviations: BMI, body mass index; WC, waist circumference; WHtR, waist-to-height ratio; BRI, body roundness index; CI, confidence interval; OR, odds ratio

[a] Q1 was <3.12, Q2 was 3.12 to <3.92, Q3 was 3.92 to <4.82, Q4 was ≥4.82

Table S10. Logistic Regression Analyses on detailed obesity category

Characteristics	Overall		Females		Males	
	**OR (95% CI)**	***P* value**	**OR (95% CI)**	***P* value**	**OR (95% CI)**	***P* value**
WHO BMI standard						
Underweight (<18.5)	1.03 (0.85, 1.24)	0.765	0.99(0.77, 1.28)	0.963	1.20(0.97, 1.50)	0.096
Normal (18.5-24.9)	1 (Ref)		1 (Ref)		1 (Ref)	
Overweight (25-29.9)	**0.76 (0.70, 0.82)**	**<0.001**	**0.78(0.70, 0.86)**	**<0.001**	**0.73(0.62, 0.86)**	**<0.001**
Obesity Class 1(30-34.9)	**0.68 (0.57, 0.81)**	**<0.001**	**0.68(0.55, 0.85)**	**<0.001**	**0.69(0.51, 0.95)**	**0.021**
Obesity Class 2(35-39.9)	**0.65(0.43, 0.99)**	**0.044**	0.68(0.41, 1.13)	0.139	0.60(0.28, 1.30)	0.194
Obesity Class 3 (≥40)	0.92(0.26, 3.26)	0.896	1.05(0.26, 4.18)	0.944	0.25(0.03, 1.76)	0.162
P for trend		**<0.001**		**<0.001**		**<0.001**

Analyses were fully adjusted for age, gender, township, education, income, cigarette smoking, alcohol drinking, hypertension, diabetes, dyslipidemia, chronic kidney diseases, fruit/vegetable intake, red meat intake, HbA1c, HDL-C, LDL-C, Scr, and triglyceride

Abbreviations: BMI, body mass index; CI, confidence interval; OR, odds ratio

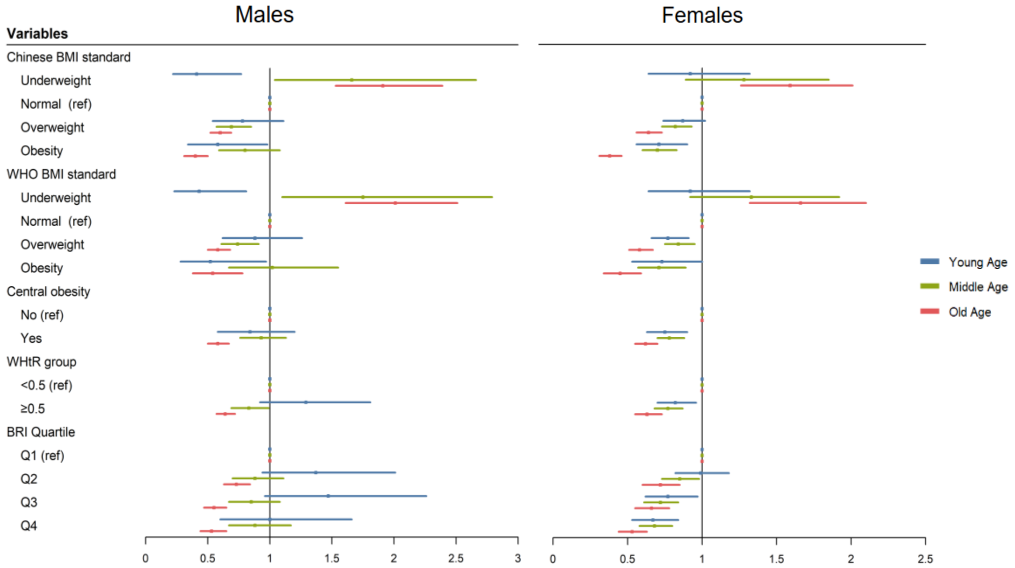

Figure S1. The gender- and age group-specific logistic regression results after full adjustment

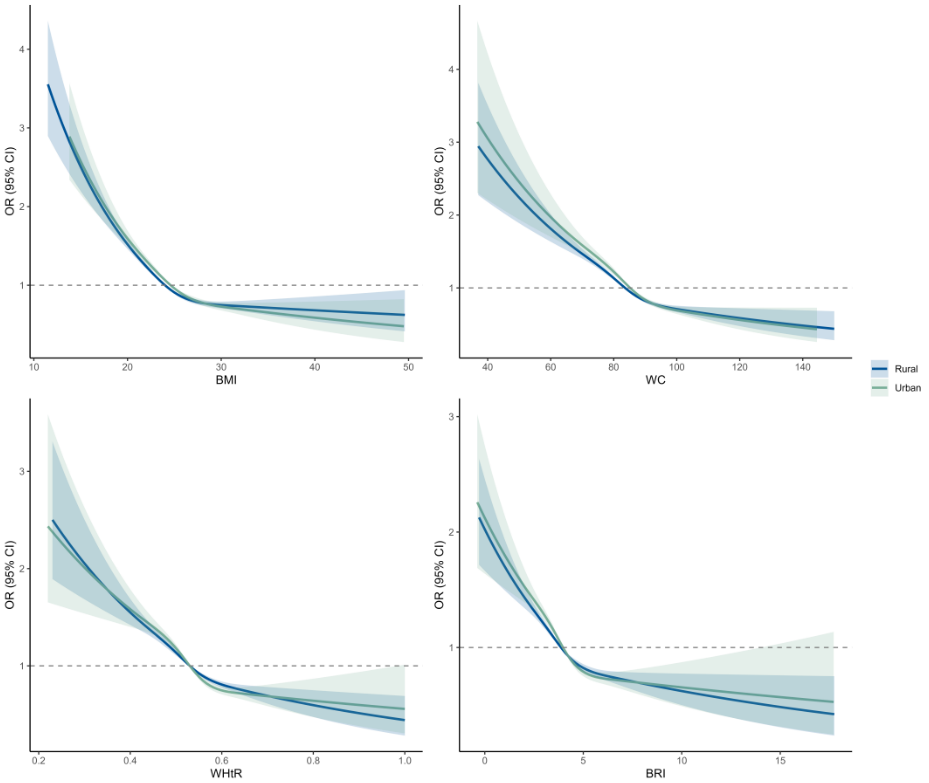

Figure S2. RCS analyses results on the association between study variables and anaemia across different townships

Analyses were fully adjusted for age, gender, township, education, income, cigarette smoking, alcohol drinking, hypertension, diabetes, dyslipidemia, chronic kidney diseases, fruit/vegetable intake, red meat intake, HbA1c, HDL-C, LDL-C, Scr, and triglyceride. Reference points for the study variables were set as their medians.

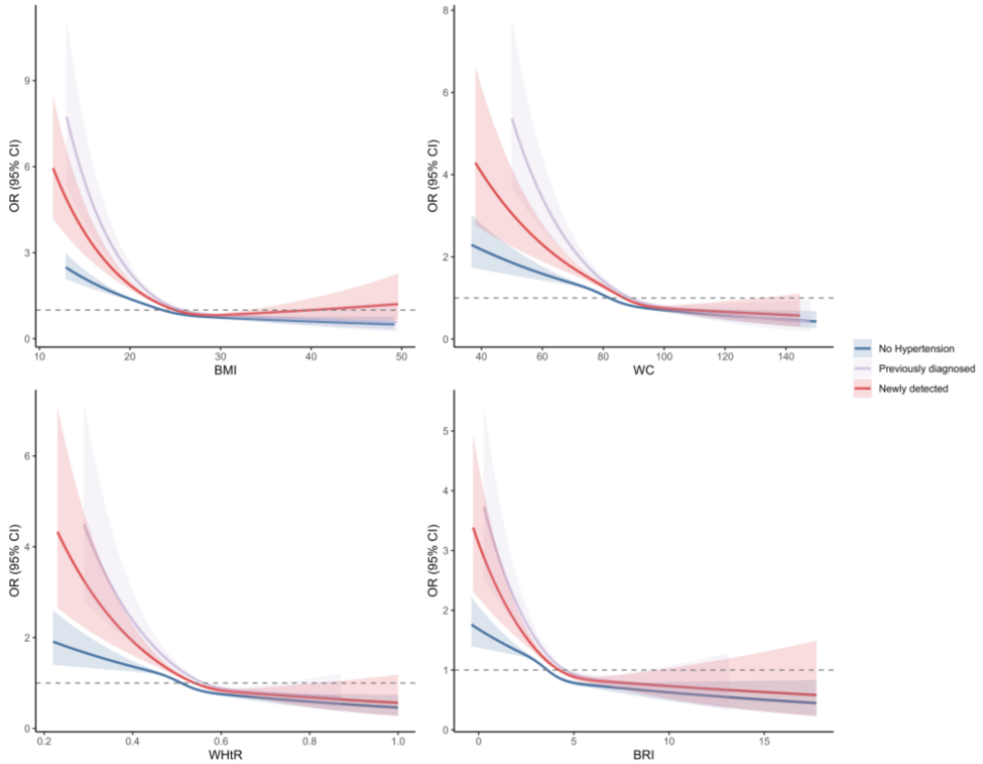

Figure S3. RCS analyses results on the association between study variables and anaemia across different hypertension status

Analyses were fully adjusted for age, gender, township, education, income, cigarette smoking, alcohol drinking, hypertension, diabetes, dyslipidemia, chronic kidney diseases, fruit/vegetable intake, red meat intake, HbA1c, HDL-C, LDL-C, Scr, and triglyceride. Reference points for the study variables were set as their medians.

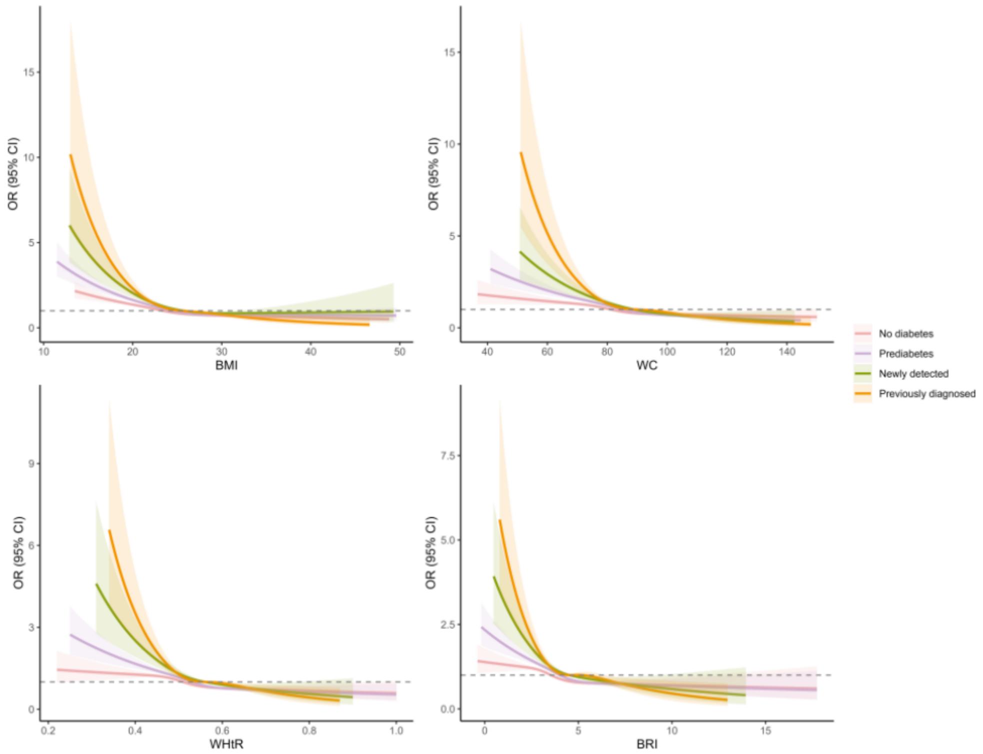

Figure S4. RCS analyses results on the association between study variables and anaemia across different diabetes status

Analyses were fully adjusted for age, gender, township, education, income, cigarette smoking, alcohol drinking, hypertension, diabetes, dyslipidemia, chronic kidney diseases, fruit/vegetable intake, red meat intake, HbA1c, HDL-C, LDL-C, Scr, and triglyceride. Reference points for the study variables were set as their medians.
